# Heterochromatin Protein 1β (HP1β) has distinct functions and distinct nuclear distribution in pluripotent versus differentiated cells

**DOI:** 10.1186/s13059-015-0760-8

**Published:** 2015-09-28

**Authors:** Anna Mattout, Yair Aaronson, Badi Sri Sailaja, Edupuganti V. Raghu Ram, Arigela Harikumar, Jan-Philipp Mallm, Kae Hwan Sim, Malka Nissim-Rafinia, Emmanuelle Supper, Prim B. Singh, Siu Kwan Sze, Susan M. Gasser, Karsten Rippe, Eran Meshorer

**Affiliations:** Department of Genetics, The Institute of Life Science and The Edmond and Lily Center for Brain Sciences The Hebrew University of Jerusalem, Jerusalem, 91904 Israel; Research Group Genome Organization & Function, Deutsches Krebsforschungszentrum (DKFZ) and BioQuant, Im Neuenheimer Feld 280, Heidelberg, Germany; School of Biological Sciences, Nanyang Technological University, 60 Nanyang Drive, Singapore, Singapore; Present address: Department of Natural Sciences and Psychology, Liverpool John Moores University, Byrom Street, Liverpool, L3 3AF UK; Friedrich Miescher Institute for Biomedical Research, Maulbeerstrasse 66, 4058 Basel, Switzerland

## Abstract

**Background:**

Pluripotent embryonic stem cells (ESCs) have the unique ability to differentiate into every cell type and to self-renew. These characteristics correlate with a distinct nuclear architecture, epigenetic signatures enriched for active chromatin marks and hyperdynamic binding of structural chromatin proteins. Recently, several chromatin-related proteins have been shown to regulate ESC pluripotency and/or differentiation, yet the role of the major heterochromatin proteins in pluripotency is unknown.

**Results:**

Here we identify Heterochromatin Protein 1β (HP1β) as an essential protein for proper differentiation, and, unexpectedly, for the maintenance of pluripotency in ESCs. In pluripotent and differentiated cells HP1β is differentially localized and differentially associated with chromatin. Deletion of HP1β, but not HP1α, in ESCs provokes a loss of the morphological and proliferative characteristics of embryonic pluripotent cells, reduces expression of pluripotency factors and causes aberrant differentiation. However, in differentiated cells, loss of HP1β has the opposite effect, perturbing maintenance of the differentiation state and facilitating reprogramming to an induced pluripotent state. Microscopy, biochemical fractionation and chromatin immunoprecipitation reveal a diffuse nucleoplasmic distribution, weak association with chromatin and high expression levels for HP1β in ESCs. The minor fraction of HP1β that is chromatin-bound in ESCs is enriched within exons, unlike the situation in differentiated cells, where it binds heterochromatic satellite repeats and chromocenters.

**Conclusions:**

We demonstrate an unexpected duality in the role of HP1β: it is essential in ESCs for maintaining pluripotency, while it is required for proper differentiation in differentiated cells. Thus, HP1β function both depends on, and regulates, the pluripotent state.

**Electronic supplementary material:**

The online version of this article (doi:10.1186/s13059-015-0760-8) contains supplementary material, which is available to authorized users.

## Background

Embryonic stem cells (ESCs), derived from the blastocyst-stage embryo, are capable of generating all cell types of the mammalian body (pluripotency) and of maintaining the capacity for indefinite self-renewal without compromising their genomic integrity. This unique duality makes them an attractive system for potential regenerative medicine and cell therapies, but also for differentiation studies in vitro and for modeling diseases. Their potential to form embryonic cell types suggests that they have unique and flexible epigenetic features and chromatin organization, two features that have attracted considerable attention in recent years [1–4].

Indeed, chromatin proteins were shown to be more dynamically associated with chromatin in ESCs than in differentiated cells [5, 6]. In addition, the nuclear lamina protein lamin A/C (LMNA), which is barely detectable in undifferentiated ESCs, is partly responsible for the restriction of chromatin plasticity during early differentiation [5]. Chromatin modifiers, such as the histone H3 lysine 9 (H3K9) methyltransferase G9a, histone deacetylases, and chromatin remodelers (e.g., CHD1 and SMARCD1) [5, 7–9], work together with lamin A/C to reduce nuclear plasticity. The genomes of ESCs also have low levels of DNA methylation, particularly when the cells are held in an undifferentiated ‘naïve’ state resembling the inner cell mass [10, 11]. Consistently, pluripotent cells are enriched for histone modifications associated with active chromatin, and tend to be depleted for heterochromatin-associated modifications, such as H3K9me3 [12–15]. Finally, we note that the undifferentiated ESC nucleus itself shows less spatial organization than in differentiated cells. For instance, condensed heterochromatin, which can be observed by both light and electron microscopy, is less frequently observed [16–18], and Heterochromatin Protein 1 (HP1)α-enriched heterochromatin foci are less compact and less numerous in ESCs [2, 13].

In mammals, the HP1 family includes three protein isoforms, HP1α (CBX5), HP1β (CBX1), and HP1γ (CBX3), encoded by the genes *Cbx5*, *Cbx1* and *Cbx3*, respectively. HP1 proteins were originally identified in *Drosophila* as structural proteins of heterochromatin and were shown to be important regulators of heterochromatin-mediated gene silencing [19, 20]. Later, the functions of HP1 proteins were extended to include additional cellular processes, such as transcriptional activation and elongation, sister chromatid cohesion, chromosome segregation, telomere maintenance, DNA repair, and RNA splicing [21–27]. It is not known how these activities are distributed among the different higher eukaryotic HP1 variants.

All HP1 proteins contain two conserved domains, the chromo-domain and the chromoshadow domain, separated by a less structured hinge region. The chromo-domain can recognize and bind the H3K9me2/me3 histone marks, which are frequently associated with transcriptional repression [28, 29]. The chromoshadow domain is required for dimerization and interaction with other proteins that share a PXVXL motif [30]. As mentioned above, HP1 isoforms have both overlapping and distinct cellular functions, and their subcellular localizations are dissimilar in some cells. Specifically, mammalian HP1α and HP1β primarily associate with dense heterochromatic and silenced genomic regions in differentiated cells, while HP1γ mainly localizes to euchromatic regions, often being associated with transcriptionally active regions [31–33]. HP1 isoform functions are not interchangeable, given that the inactivation of HP1β in mice leads to a defective development of neuromuscular junctions and cerebral cortex as well as perinatal lethality, despite the presence of HP1α and HP1γ [34]. However, little is known about how the different isoforms are regulated.

As mentioned above, a growing number of chromatin-related factors are implicated in either the maintenance of pluripotency or the differentiation of ESCs. Examples include chromatin remodeling proteins [8, 35–37], histone modifying enzymes [38–44], histone variants [45–50], and HP1γ [15]. Reducing HP1γ levels in ESCs under differentiating conditions was shown to enhance differentiation, and to improve the reprogramming of somatic cells into induced pluripotent stem cells (iPSCs) [15, 51]. Here we show that another member of the heterochromatin protein family, HP1β, is necessary to maintain proper differentiation in differentiated cells, but surprisingly, it is also necessary to maintain pluripotency in ESCs in normal conditions, unlike HP1γ. This is not the case for HP1α. In addition, unlike somatic and differentiated cells, HP1β does not localize primarily to heterochromatic chromocenters in ESCs, but rather assumes a diffuse nuclear localization. It is highly expressed in ESCs, and on chromatin it is enriched in genic, mostly exonic regions. Importantly, loss of HP1β results in premature, spontaneous differentiation along with misregulation of several pluripotency factors and developmental genes. The fact that HP1β exhibits two distinct nuclear localizations and plays nearly opposing roles at two states of differentiation (pluripotent versus differentiated cells) argues that a single HP1 protein can assume strikingly distinct roles as a function of cell differentiation. This significantly embellishes previous concepts of HP1 function, which assigned distinct localization and function to different HP1 isotypes.

## Results and discussion

### HP1β, but not HP1α, is essential to maintain pluripotency and cell proliferation in ESCs

**Fig. 1:**
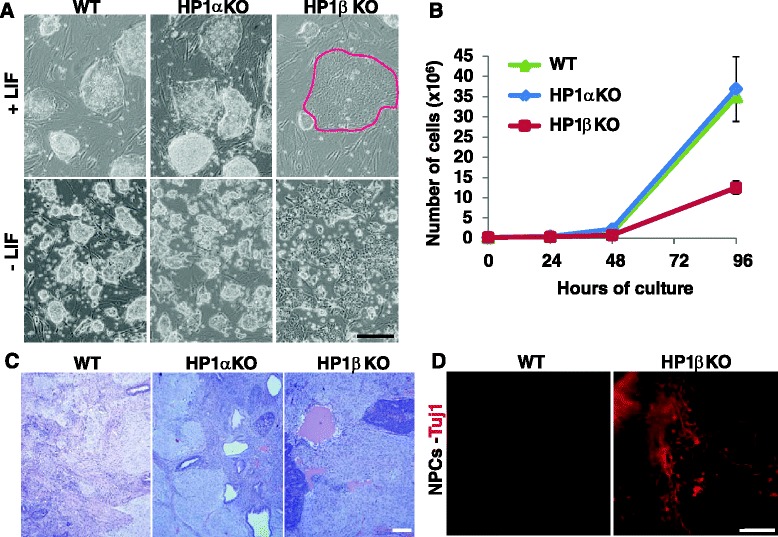
HP1β is essential to maintain pluripotency and cell proliferation in ESCs. **a** Premature differentiation of HP1β^−/−^ ESCs. Shown are WT (*left*), HP1α^−/−^ (*middle*), and HP1β^−/−^ (*right*) ESCs grown in the presence (*top*) or absence (*bottom*) of LIF. WT and HP1α^−/−^ ESCs maintain normal growth and colony morphology whereas the HP1β^−/−^ ESCs tend to spontaneously differentiate and form flat colonies (*red outline*). Scale bar = 200 μm. **b** Cell proliferation assay. Proliferation rate is reduced in HP1β^−/−^ ESCs, but unaltered in HP1α^−/−^ ESCs. **c** Histological analyses of teratomas formed by WT, HP1α^−/−^ and HP1β^−/−^ESCs. The three germ layers were observed in all teratomas but neuroectodermal differentiation (*dark blue*) appeared particularly enriched in the HP1β^−/−^ cells. Scale bar = 200 μm. **d** Accelerated neuronal differentiation in directed differentiation of HP1β^−/−^ ESCs. Neuronal progenitor cells (NPCs) from WT and HP1β^−/−^ ESCs were immunostained with Tuj1. Scale bar = 25 μm

In order to determine whether HP1α and/or HP1β isoforms have any role in stem cell pluripotency and early differentiation, we took advantage of the recently generated HP1α^−/−^ and HP1β^−/−^ knockout (KO) mice and of the derived pluripotent ESCs, the differentiated embryoid bodies (EBs), and the mouse embryonic fibroblast (MEF) cells from these KO strains [34, 52]. To explore whether HP1α or HP1β has a specific function in pluripotent/undifferentiated cells, we analyzed the morphology of HP1α^−/−^ and HP1β^−/−^ ESCs, their cell growth, and differentiation potential compared with their wild-type (WT) counterparts at identical passages under identical conditions. To validate the KO clones and the specificity of the HP1α and HP1β antibodies, we verified the absence of the specific HP1 protein in the appropriate cell line, using immunofluorescence (IF) and western blots (Figure S1a, b in Additional file 1). As we cultured the KO ESCs, we noticed unexpectedly that whereas WT and HP1α^−/−^ ESCs displayed normal colony morphology, most of the HP1β^−/−^ ESCs did not form the usual compact three-dimensional colonies. They tended instead either to differentiate spontaneously or to remain very small (Fig. 1a). This was observed both in the presence of leukemia inhibitory factor (LIF), which maintains ESCs in their undifferentiated state, and in its absence, where the effect was more pronounced. We also observed that the HP1β^−/−^ ESCs differentiated faster than WT and/or HP1α^−/−^ ESCs upon LIF depletion (Fig. 1a, lower panel). The same was true when differentiation was induced by retinoic acid (RA; data not shown). Finally and importantly, HP1β^−/−^ but not HP1α^−/−^ ESCs displayed significantly reduced growth rates (Fig. 1b), indicating a reduced capacity for self-renewal.

We next tested the differentiation potential of HP1α^−/−^ and HP1β^−/−^ ESCs. To this end, we performed a teratoma assay, which involves injecting HP1α^−/−^, HP1β^−/−^, and WT ESCs under the skin of SCID mice. Three weeks later the resulting teratomas were analyzed by histology. We detected increased neuroectoderm formation in teratomas derived from HP1β^−/−^ ESCs, although all three germ layers were present in all the teratomas of all cell lines tested (Fig. 1c). To validate this observation, we performed directed differentiation of WT and HP1β^−/−^ ESCs into neuroectoderm in vitro. HP1β^−/−^ ESCs displayed accelerated neuronal differentiation, as judged by morphology and increased Tuj1-positive cells (Fig. 1d). Together, these results argue that the absence of HP1β in ESCs compromises the maintenance of pluripotency and cell proliferation, and increases neuronal differentiation both in vitro and in vivo. This suggests that HP1β negatively regulates neuronal differentiation in pluripotent cells and is thereby required to maintain pluripotency. We confirmed the results for the KO ESCs by RNA interference for HP1β which, similarly, led to premature differentiation (Figure S2b in Additional file 2).

HP1β is 100 % conserved between mouse and human, and mouse HP1β and HP1α are 63 % identical (and 79 % similar). It was of interest, therefore, to examine the effects of HP1β loss on overall chromatin organization. First, visualizing pericentromeric heterochromatin by DAPI, we note that the absence of HP1β had no significant impact on the global structure of pericentromeric heterochromatin domains in ESCs (Figure S1c in Additional file 1), nor did loss of HP1α (Figure S1c in Additional file 1). In addition, H3K9me3 staining of pericentromeric heterochromatic foci, as shown by the overlap with the DAPI staining in MEFs and ESCs, was also not altered in HP1α^−/−^ and HP1β^−/−^ ESCs compared with their WT counterparts (Figure S1c in Additional file 1). This observation is in line with previous reports in differentiated 3T3 mouse fibroblasts [53].

Using a more quantitative approach, we monitored fluorescence recovery after photobleaching (FRAP) for H1-GFP, as an indicator for chromatin plasticity [5]. This is used to monitor the impact of HP1β depletion on general chromatin proteins, as previously reported for CHD1 in euchromatic regions [8]. However, H1 protein dynamics in WT and HP1β^−/−^ ESCs were not significantly different (Figure S1d in Additional file 1). Indeed, as described below, HP1β itself is relatively poorly associated with chromatin in ESCs (see Fig. 7). Finally, to test whether the reduced capacity for self-renewal of HP1β^−/−^ ESCs (Fig. 1b) could be explained by defects in chromosome segregation during mitosis, we monitored metaphase and anaphase cells in HP1α^−/−^, HP1β^−/−^ and WT ESCs (Figure S2a in Additional file 2). No defects, such as DNA bridges, were detected in any of the anaphase ESCs, although H3K9me3 has been described to be important for chromosome segregation [29]. In addition, H3K9me3 staining in these cells was perfectly localized mainly at pericentromeric regions, as expected (Figure S2a in Additional file 2). This suggests that chromosome segregation can occur normally in HP1β^−/−^ ESCs.

### HP1β regulates developmental genes and pluripotency factors in ESCs

**Fig. 2:**
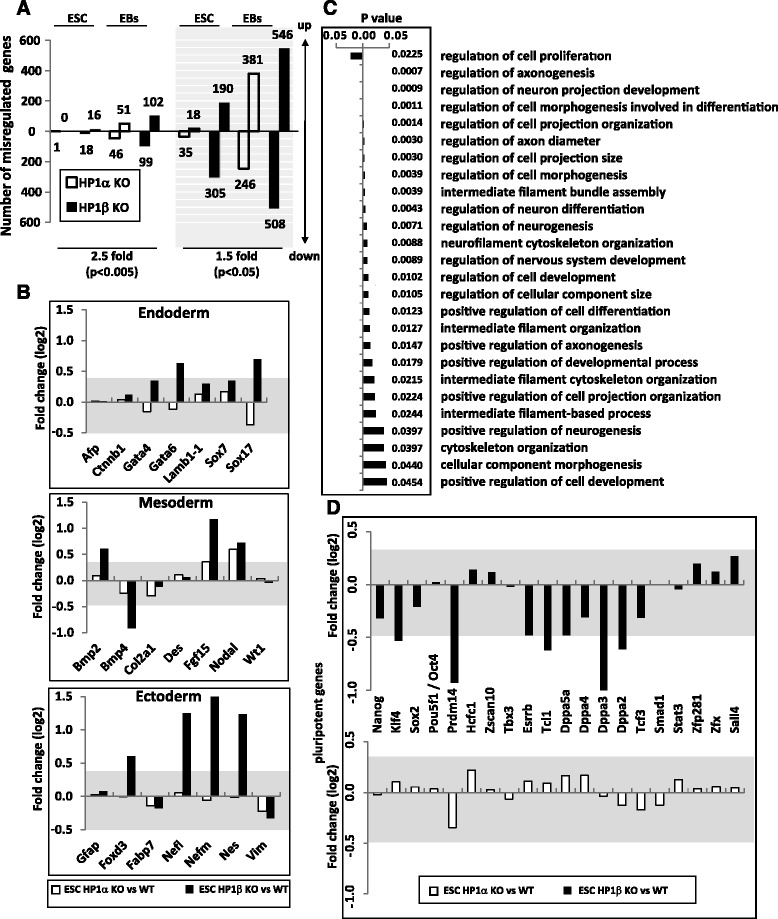
HP1β regulates developmental genes and pluripotency factors in ESCs. **a** Number of misregulated genes in HP1α^−/−^ (*empty bars*) and HP1β^−/−^ (*filled bars*) ESCs and EBs compared with WT cells at a 0.005 (*left*) or 0.05 (*right*, *shaded*) confidence level, which correspond to 2.5- and 1.5-fold change, respectively. **b** Expression fold change in HP1α^−/−^ (*empty bars*) and HP1β^−/−^ (*filled bars*) ESCs compared with WT cells of developmental marker genes representative of endoderm (*top*), mesoderm (*middle*) and ectoderm (*bottom*). The *shaded grey area* represents fold changes which are not statistically significant. **c** Gene Ontology analysis of biological processes affected in the HP1β^−/−^ ESCs. Biological processes where bars in the graph go to the left of zero (here, only “regulation of cell proliferation”) are those affected significantly by genes downregulated in HP1β^−/−^ ESCs, whereas those where bars in the graph go to the right of zero are those significantly affected by genes upregulated in HP1β^−/−^ ESCs. The actual *p* values are shown for each biological process. The list of the misregulated genes was analyzed according to their functional annotation and the biological processes they belong using the Database for Annotation, Visualization and Integrated Discovery (DAVID)*.* **d** Expression fold change in HP1α^−/−^ (*empty bars*) and HP1β^−/−^ (*filled bars*) ESCs compared with WT cells of key pluripotency factors. The grey area represents fold changes which are not statistically significant

Given the strong phenotypic effect of HP1β deletion on pluripotency, and the absence of change in chromatin organization, we next looked for effects on the level of gene expression. Using Affymetrix whole transcriptome microarrays (GSE65121), we analyzed transcription profiles of WT, HP1α^−/−^ and HP1β^−/−^ ESCs in duplicates, and after EB differentiation for 7 days. EBs are known to undergo non-directed differentiation and cell specification into the three germ lineages (endoderm, ectoderm, and mesoderm). To ensure that neither MEFs nor spontaneously differentiating cells contaminated our ESC preparations, we sorted the pluripotent SSEA1-positive cells from all ESC types using magnetic beads and a column-based method. This is particularly important in the case of HP1β^−/−^ ESCs, since, as noted above, these cells tend to spontaneously differentiate. Using a threshold of 1.5-fold change in mRNA level (corresponding to *p* < 0.05; Figure S3a in Additional file 3) comparing mutant and WT ESCs and EBs, we found that the loss of HP1β resulted in the misregulation of 495 and 1054 genes in ESCs and EBs, respectively. The loss of HP1α, on the other hand, had a more subtle effect in both ESCs and EBs, with 53 and 627 genes altered, respectively (Fig. 2a, right). When a stringent cutoff of 2.5-fold in transcription level was used (corresponding to *p* < 0.005; Figure S3a in Additional file 3), only one gene passed the threshold in the HP1α^−/−^ ESCs, and 97 genes did in the corresponding EBs. In contrast, the HP1β^−/−^ ESCs had 34 genes in the undifferentiated ESCs and 201 in the corresponding EBs that were at least 2.5-fold misregulated (Fig. 2a, left). Changes in gene expression were validated in both ESCs and EBs using quantitative RT-PCR (qRT-PCR) for several genes (*r*^*2*^ > 0.8 between the two methods; Figure S3b, c in Additional file 3). We conclude that HP1β has a far more significant effect on gene expression in both ESCs and EBs than HP1α.

We next examined the misregulation of established lineage markers in ESCs and found that, once again, HP1α deficiency had a relatively mild effect, with none of the selected markers showing a significant change (Fig. 2b). In contrast, depletion of HP1β resulted in significant changes in the expression of genes from all lineages examined, including endoderm, mesoderm, ectoderm, and trophoectoderm (Eomes). The most pronounced effect was again in neuroectoderm lineage markers, where significant overexpression of a related set of genes was detected (Fig. 2b). This correlates well with the changes observed in protein levels of neuroectodermal markers and with the effect of HP1β deletion on teratoma formation (Fig. 1c, d). Consistently, Gene Ontology (GO) analysis for the genes upregulated >2.5-fold in the HP1β^−/−^ ESCs revealed a significant enrichment in categories reflecting neuronal differentiation and cell proliferation (Fig. 2c). In contrast, the effect of HP1α deletion was again insignificant, even when the more relaxed threshold of 1.5-fold was used. Importantly, loss of HP1β in ESCs also led to a significant downregulation of key pluripotency factors (Fig. 2d), a fact that may explain the partial loss of pluripotency characteristics of those cells (morphology, growth rate, etc.). This is unlike the loss of HP1α (Fig. 2d) and unlike depletion of HP1γ, which show normal expression of pluripotency markers [15, 51]. In summary, we find that the loss of HP1β in ESCs downregulates the expression of pluripotency factors and skews the expression of developmental genes. This correlates with premature ESC differentiation, particularly along the neuroectodermal lineage. Such effects are unique to HP1β.

**Fig. 3:**
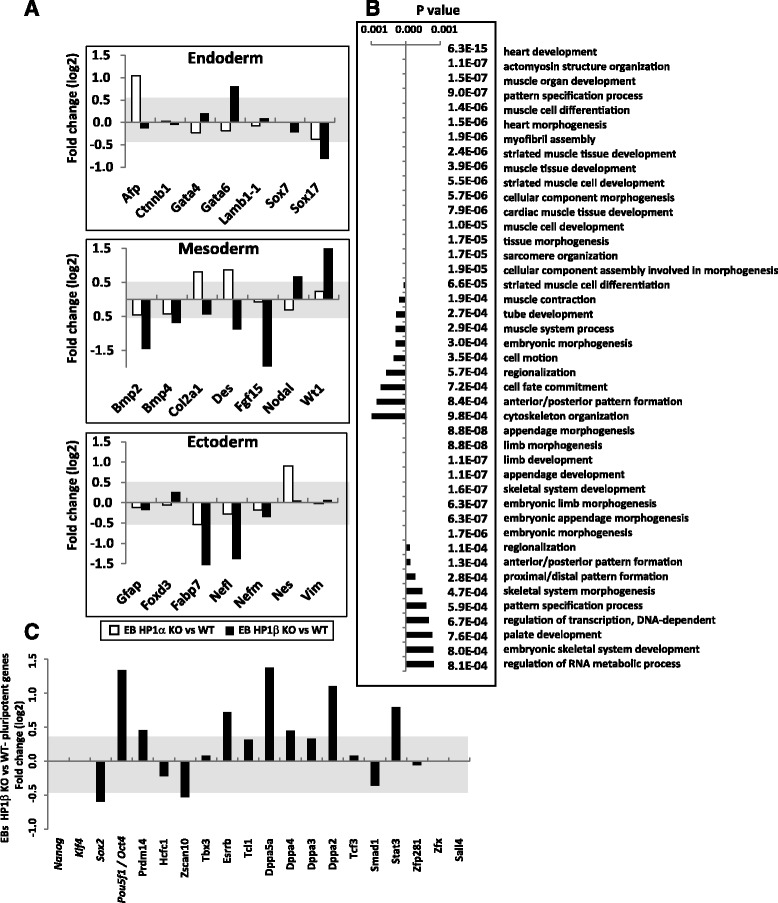
HP1β is important for mesodermal differentiation of embryoid bodies. **a** Relative fold change of developmental genes of the three germ layers in EBs derived from HP1α KO ESCs (*empty bars*) and EBs derived from HP1β KO ESCs (*filled bars*). Upregulated genes are depicted above the line at zero, and downregulated genes below it. The grey area represents fold changes which are not statistically significant. **b** GO analysis of biological processes affected in HP1β KO EBs. Biological processes where the bars in the graph go to the left of zero are those affected significantly by genes downregulated in HP1β^−/−^ EBs, whereas the those where the bars go to the right of zero are those significantly affected by genes upregulated in HP1β^−/−^ EBs. The actual *p* values are shown for each biological process. **c** Relative fold change of key pluripotent factors for EBs derived from HP1β KO ESCs. Upregulated genes are depicted above the line at zero and downregulated genes below it. The grey area represents fold changes which are not statistically significant

**Fig. 4:**
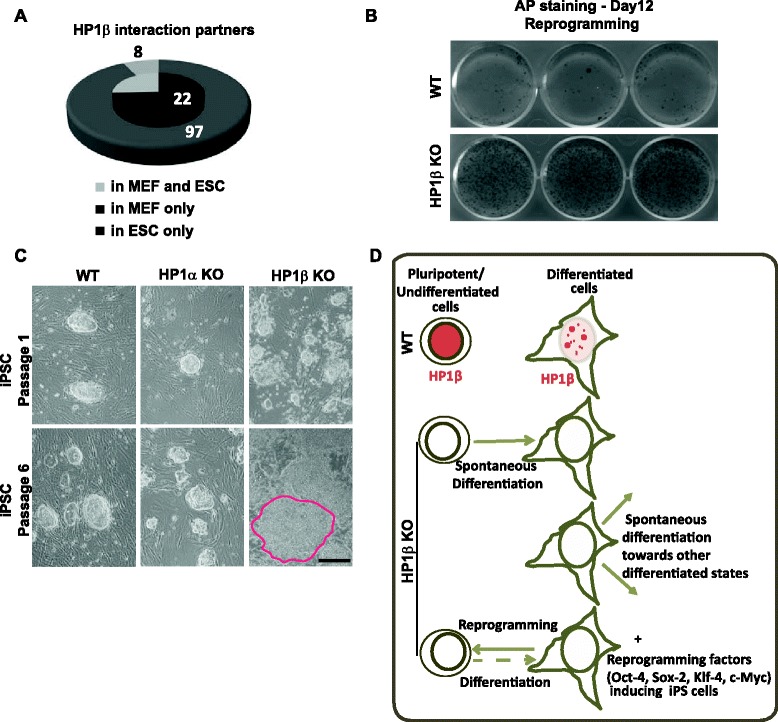
HP1β has different interacting partners and functions in pluripotent and differentiated cells. **a** The number of HP1β interacting partners identified by liquid chromatography-tandem mass spectrometry in MEFs, and in ESCs excluding hits found in control samples. Hits identified in both cell types are indicated. **b** Reprogramming experiments. Alkaline phosphatase (*AP*) staining of iPSCs induced from WT MEFs and HP1β KO MEFs after 12 days of reprogramming in identical conditions. **c** Phase contrast images of WT (*left*), HP1α KO (*middle*) and HP1β KO (*right*) iPSC colonies cultured in standard conditions at passage 1 (*top*) or passage 6 (*bottom*) after isolation from the reprogramming plate. HP1β KO iPSCs gave rise to flat and spontaneously differentiating cells (*red outline*), similar to HP1β KO ESCs. Scale bar = 200 μm. **d** Graphic summary of HP1β localization and function in pluripotent versus differentiated cells. In WT cells, HP1β is highly expressed and diffuse in ESC and iPSC nuclei whereas it decreases in differentiated cells and associates mostly with pericentric heterochromatin. HP1β KO pluripotent cells do not maintain a proper pluripotent state and tend to differentiate spontaneously; differentiating cells lacking HP1β display skewed differentiation, and reprogramming is facilitated in the absence of HP1β

To determine whether HP1β KO also affects later stages of differentiation, we compared the transcriptional profiles from 7-day-old EBs originating from WT, HP1α^−/−^ and HP1β^−/−^ ESCs. As in earlier stages, loss of HP1α had a mild effect on gene expression, and it subtly, but significantly, altered lineage markers of the three germ layers. Loss of HP1β, on the other hand, had a particularly robust effect on mesodermal lineage markers. For instance, loss of HP1β led to the downregulation of Bmp2, Bmp4, Des, and Fgf15 (Fig. 3a). GO analysis on the altered genes (using a threshold of 2.5-fold) in HP1β^−/−^ EBs indicated strong effects on heart and muscle development (Fig. 3b), consistent with mesodermal differentiation defects. This is consistent with the fact that modulation of the HP1β protein level has been found to impair MyoD target gene expression and muscle terminal differentiation [33]. Therefore, whereas the differentiation of HP1β^−/−^ ESCs was skewed towards neuroectoderm, HP1β^−/−^ EBs were skewed away from proper mesoderm formation. Interestingly, a relatively high number of actin, myosin, and related proteins, which we found as interacting partners of HP1β in differentiated cells (see below and Fig. 4), were found to be both up- and downregulated (GO category “actomyosin structural organization”) in the HP1β KO cells (Fig. 3b). Finally, several pluripotency genes, including Nanog, Oct4, Esrrb, Dppa2, Dppa5a, and Stat3 failed to be correctly downregulated in the differentiated HP1β^−/−^ EBs compared with WT EBs (Fig. 3c). It is important to point out that pluripotency factors are downregulated in HP1β^−/−^ ESCs but upregulated in the differentiating HP1β^−/−^ EBs. This result, together with the distinct effects that HP1β elimination has on ESCs and EBs, suggests that HP1β influences gene expression in opposite directions — or at the very least triggers distinct pathways of gene control — in pluripotent versus differentiated cells.

### HP1β has different interacting partners in pluripotent and differentiated cells

Because HP1β has very distinct and contrasting effects on gene regulation in pluripotent versus differentiated cells, we checked whether HP1β is associated with different protein complexes in the two cell states. To examine HP1β’s interacting partners in pluripotent and differentiated cells, we immunoprecipitated the endogenous HP1β from both ESC and MEF extracts, and used liquid chromatography-tandem mass spectrometry (LC-MS/MS) to examine co-precipitating proteins. This allowed us to avoid potential artifacts due to overexpression or the addition of tags. Experiments were performed in two biological replicates and non-specific interactions were eliminated using anti-green fluorescent protein (anti-GFP) as a negative control. Several HP1β interaction partners were common to both ESCs and MEFs, including hnRNPH2, hnRNPA0, Rundc2a, Eif4enif1 and histone H2B (Fig. 4a; Additional file 4). However, the large majority of HP1β’s interaction partners differed between the two cell types (Fig. 4a), suggesting that the recovery is not a product of contamination. Moreover, the number of identified HP1β interacting partners overall was considerably lower in ESCs than in MEFs (30 versus 105 proteins; Additional file 4). Whereas it is impossible to infer function from simple immunoprecipitation, the fact that we recovered different sets of interacting partners is consistent with a distinct function for HP1β in differentiated cells.

### HP1β restricts reprogramming into iPSCs

The distinct effects on gene expression and the different interaction partners of HP1β in ESCs and MEFs prompted us to test its potential involvement in somatic cell reprogramming to iPSCs. To this end, we generated iPSC colonies from WT and HP1β KO MEFs by lentiviral infection expressing the four reprogramming factors Oct4, Sox2, Klf4, and cMyc. HP1β KO MEFs displayed increased reprogramming efficiency compared with WT MEFs as judged by the number of iPSC colonies generated after 12 days of reprogramming in identical conditions by alkaline phosphatase staining (Fig. 4b). This again suggested that, like HP1γ [15], HP1β helps maintain a proper differentiation state in WT differentiated cells by inhibiting efficient reprogramming. Indeed, heterochromatin reorganization was found to be one of the first steps in the rearrangement of chromatin from a somatic-like to a pluripotent-like state during the reprogramming process [14].

Importantly, and consistent with the phenotypes we observed in HP1β^−/−^ ESCs, fully reprogrammed HP1β KO iPSCs exhibit similar properties to those of HP1β^−/−^ ESCs. They tend to differentiate spontaneously and rapidly, especially in the absence of a feeder layer, losing their compact morphology after several passages (Fig. 4c). In contrast, iPSC colonies generated from HP1α KO MEFs were morphologically indistinguishable from WT iPSC colonies and HP1α KO ESCs (Fig. 4c). Taken together, our findings confirm that pluripotent cells such as ESCs and iPSCs that lack HP1β tend to differentiate spontaneously. On the other hand, HP1β^−/−^ differentiated cells could not maintain a proper differentiation state (EBs) and reprogrammed into iPSCs more easily than WT cells (MEFs) (Fig. 4d). This contrasting behavior argues that HP1β has distinct roles at different stages of differentiation. HP1β maintains pluripotency in ESCs, while in differentiated cells it helps maintain the differentiated state.

### HP1β is highly expressed and diffuse in nuclei of pluripotent cells

**Fig. 5:**
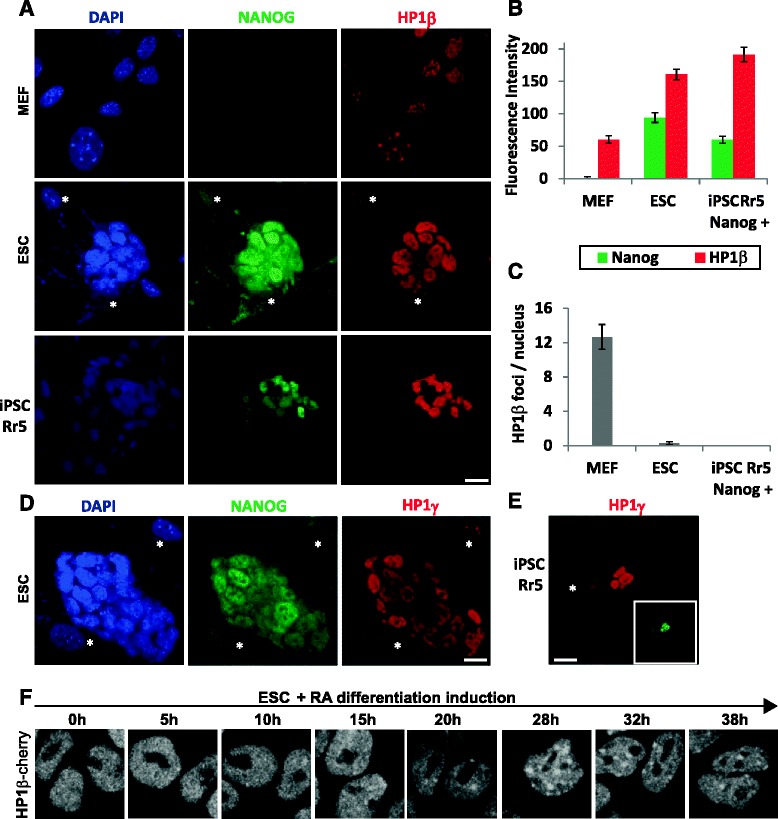
HP1β is highly expressed and diffuse in nuclei of pluripotent cells. **a** Confocal images of MEFs (*top*), R1 ESCs (*middle*) and Rr5 iPSCs (*bottom*) immunostained for Nanog (*green*, *middle*), HP1β (*red*, *right*) and counterstained with DAPI (*blue*, *left*). *Asterisks* indicate examples of MEFs used as a feeder layer in the culture of the pluripotent cells. **b** Quantification of the fluorescence intensities of Nanog (*green bars*) and HP1β (*red bars*) for the three cell types (n ≥ 26). Nanog is used as a marker for pluripotent cells; the fluorescence intensity of the background intensity was subtracted. **c** Number of HP1β foci in the different cell types. Error bars in (**b**) and (**c**) represent standard error of the mean. **d** Confocal images of R1 ESCs immunostained for Nanog (*green*, *middle*), HP1γ (*red*, *right*) and counterstained with DAPI (*blue*, *left*). **e** Confocal images of Rr5 iPSCs immunostained for HP1γ (*red*) and Nanog (*inset*, *green*). *Asterisks* indicate feeder layer MEF cells in (**d**) and (**e**). Scale bars for (**a**–**e**) = 15 μm. **f** Time lapse spinning disk confocal images of ESCs expressing the endogenous HP1β fused to mCherry induced to differentiate with 1 μM of retinoic acid (*RA*) for 40 hours (see also Additional file 7 for a video)

We next asked how this can be achieved. Is there dissimilar expression and/or localization of HP1β in the different cell states? Indeed, by indirect immunofluorescence [14], we scored an approximately threefold higher expression level of HP1β in pluripotent nuclei of mouse Rr5 iPSCs [14] and R1 ESCs over that in MEFs (Fig. 5a, b). The Rr5 iPSC line contains both fully and partially reprogrammed iPSCs with otherwise similar properties (i.e., morphology, size, proliferation rate, nuclear volume) [14], conveniently enabling us to compare these two cell populations in the same field of view using Nanog staining as a marker of pluripotency. Only the “fully” reprogrammed and pluripotent Rr5 iPSCs showed high levels of HP1β, arguing that elevated HP1β levels are truly linked to the pluripotent state, and do not simply reflect proliferation rate or cell size. The MEF feeder layer (some of which are marked by asterisks in Fig. 5), which is used to maintain the undifferentiated state of pluripotent cells, provided us with an additional internal control, in the same image field for HP1β staining. We also confirmed that HP1β is present at higher levels in ESCs than in MEFs by western blotting extracts from mouse ESCs and MEFs (Figure S4a, b in Additional file 5 and Fig. 7c). Total levels of HP1β normalized to the amount of histone H3 shows an enrichment in ESCs of about threefold compared with MEFs (Figure S4b in Additional file 5), consistent with fluorescence intensity. Finally, we observed a slight, but reproducible, drop in HP1β levels after 7 days of ESC differentiation towards EBs (Figure S4c in Additional file 5).

We compared our results with previous reports and with publicly available gene expression datasets [54, 55] to ensure that this variation is broadly observed, even at the transcriptional level. Consistent with our findings, the Amazonia dataset [56] shows higher HP1β expression levels in human pluripotent cells compared with all other differentiated cell types (Figure S4d in Additional file 5). In previous datasets, HP1β had a threefold higher level in undifferentiated mouse ESCs over 7-day-old neuronal progenitor cells (NPCs) derived from those ESCs by in vitro differentiation [18]. HP1γ also displayed approximately threefold higher levels in ESCs compared with NPCs, in contrast to HP1α, which was only ~1.5 fold higher in the undifferentiated cells. The fact that HP1β and HP1γ levels decrease more sharply than HP1α levels upon differentiation supports the results we obtained by immunofluorescence on pluripotent and differentiated cells (Figs. 5d-e and 7e for HP1γ; and [14] for HP1α).

Akin to other chromatin proteins, the localization of the HP1 isoforms may be more important than their absolute levels. In support of this, we found that HP1β has a diffuse nucleoplasmic staining pattern in the nuclei of iPSCs and ESCs, in stark contrast to the characteristic heterochromatic foci found in the nuclei of differentiated MEFs (Fig. 5a, c; see Figure S6a in Additional file 6 for shorter exposure). This phenomenon was also true for HP1γ (Fig. 5d, e), but was not the case for HP1α. Whereas HP1α is somewhat diffuse in the nuclei of pluripotent cells, it also clearly labels heterochromatic foci [6, 14]. We quantified these differences by counting the average number of HP1β-positive foci in each cell type. We scored, on average, 12.2 ± 2.4 HP1β foci per nucleus in MEFs and 0.1 ± 0.4 in either fully reprogrammed iPSCs or ESCs (Fig. 5c). These observations were reproducible under different conditions, and are consistent with previous studies which showed fewer HP1β heterochromatic foci in E14 mouse ESC line (4 foci per ESC) than in a more differentiated state (11 HP1β foci per cell [57]). We note that the E14 ESCs displayed a lower level of histone acetylation and a diminished ability to reprogram MEFs by cell fusion than the R1 ESCs used above [58]. Consistent with the stronger pluripotency character of our R1 ESCs over E14 cells, we see that HP1β assumes a completely diffuse pattern in the nucleoplasm of R1 ESCs, while it was partially accumulated at heterochromatin foci in E14 ESCs (Figure S6b in Additional file 6).

A final confirmation that HP1β changes localization during differentiation came from the use of an endogenously tagged fluorescent protein library (our own unpublished resource), in which HP1β is endogenously fused with the mCherry fluorescent protein. By scoring HP1β localization in living cells we can avoid potential artifacts of fixation or overexpression. Spinning disk time lapse imaging of ESC differentiation showed that HP1β has a diffuse pattern in undifferentiated cells, which transitions to HP1β focus accumulation. This occurred within 24–36 hours, at which point all cells displayed some degree of HP1β foci (≥1–2 foci per cell; Fig. 5f; Additional file 7). Taken together, we conclude that HP1β is more highly expressed and has a diffuse subnuclear localization in pluripotent stem cells, whereas it becomes heterochromatin-enriched in differentiated cells, consistent with the different roles it has in the two cell states.

### HP1β is enriched within genes in pluripotent cells

**Fig. 6:**
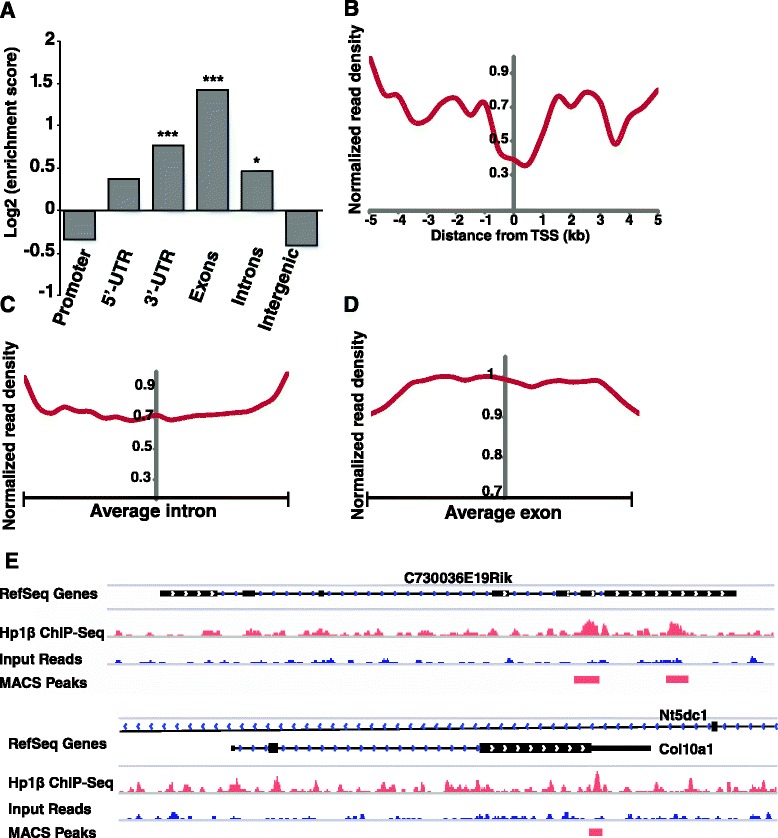
HP1β is enriched within genes in pluripotent cells. **a** ChIP-Seq enrichment scores for HP1β in the indicated genomic regions. HP1β is highly enriched within exons (*** p < 0.0001, * *p* < 0.005, permutation test). Note promoter depletion. *UTR* untranslated region. **b** Average HP1β distribution (over all genes) around the transcription start site (*TSS*). **c** HP1β distribution within an average intron. Introns are overall enriched for HP1β but depleted when compared with exons. **d** HP1β distribution within an average exon. **e** HP1β is localized in gene bodies and exons. Examples of HP1β ChIP-Seq signal in genes and exons. RefSeq gene annotation is shown in the *top row*; below, HP1β ChIP-Seq read signals are shown in *red* and input read signals are shown in *blue*. MACS peaks are shown in the *bottom row*

In order to confirm these imaging results, we investigated the distribution of HP1β genome-wide using ChIP-Seq. ChIP-Seq analysis in ESCs showed that HP1β is significantly enriched in genes, especially within exons (*p* < 10^−4^, hypergeometric test; Fig. 6a; [GEO:GSE64946]). Moreover, HP1β is largely depleted from intergenic regions in ESCs, which would normally show enrichment for heterochromatin. Moreover, HP1β was largely depleted from proximal promoters (Fig. 6a) and transcription start sites (Fig. 6b), yet showed a clear enrichment gradient that increased from introns to exons: indeed, HP1β is more strongly enriched on exons than on introns (Fig. 6c–e). This preferential association of HP1β with exons is consistent with a unique role in pluripotent cells, and suggests a potential role in exon recognition, that may coincide with histones bearing H3K36me3 [59]. Interestingly, ‘alternative splicing’ was the most highly enriched category in GO analysis performed for the HP1β-bound genes (Figure S5a in Additional file 8). These correlations suggest a potential role for HP1β in exon recognition and/or pre-mRNA processing in ESCs. This observation is in line with a recent study that showed that HP1β regulates the alternative splicing of a subset of genes in a DNA methylation-dependent manner [60], which is thought to be achieved by the recruitment of splicing factors to DNA methylated genes through HP1β [60].

Since HP1β is not known to bind methylated H3K36, we next compared the HP1β ChIP-Seq data with other existing genome-wide datasets in ESCs (Figure S5b in Additional file 8). We found significant correlation of HP1β-bound loci (*p* < < 10^−16^) with H3K36me2/me3, which is also enriched within exons [59, 61], as well as with H3K9me3 (*p* < < 10^−16^). This suggests that HP1β, while largely euchromatic and exonic in ESCs, may also be associated in some regions with H3K9me3.

To understand if the changes in gene expression in the HP1β^−/−^ ESCs resulted from transcriptional regulation by HP1β or from post-transcriptional regulation through HP1β, we tested the correlation between HP1β binding to the genome and the changes in expression level of the corresponding genes or promoter regions. Comparing the list of the misregulated genes (>1.5-fold) in the HP1β^−/−^ ESCs with the list of the promoters or gene bodies directly bound by HP1β (ChIP-Seq data), we found that promoter regions bound by HP1β do not correlate significantly with misregulation of the adjacent genes (hypergeometric *p* value > 0.9; Figure S5c in Additional file 8). The HP1β-bound exons/gene bodies selected with a mild threshold (*p* < 0.01) also had no significant correlation with upregulated transcripts in the HP1β KO ESC samples, whereas a slight correlation was found with downregulation. When a more stringent threshold was used for the HP1β-bound genes (*p* < 0.001), a higher significance level was observed for a group of 15 genes that were clearly downregulated in HP1β^−/−^ ESCs (Figure S5c–e in Additional file 8), suggesting that HP1β could potentially upregulate the transcription of this subset of genes in WT ESCs. Nonetheless, since the majority (>97 %) of HP1β-bound genes in ESCs had no change in their expression level in HP1β^−/−^ ESCs, it appears that, in pluripotent ESCs, HP1β by itself probably does not act principally by modulating transcription. Supporting this view, we found that the genes that are misregulated in HP1β KO ESCs and that are included in biological process categories such as “regulation of cell proliferation” or “regulation of cell development” (Fig. 2c; e.g., *Inpp5D*, *Ifitm3*, *Nefl*, *Nefm*, *Tnfrsf12a*) are not genes or promoter regions bound by HP1β in ESCs. Nor are pluripotency factors such as Nanog or Klf4 downregulated in HP1β KO ESCs (see below). In addition, none of the genes (listed in Figure S5e in Additional file 8) bound by HP1β and misregulated in HP1β KO ESCs seem a priori able to explain all the phenotypes observed in HP1β KO ESCs. Alternatively, HP1β may work by modulating mRNA processing or export or may serve to maintain a chromatin state that only affects gene expression at a later point in development.

### HP1β binds chromatin in a distinct manner in pluripotent and differentiated cells

**Fig. 7:**
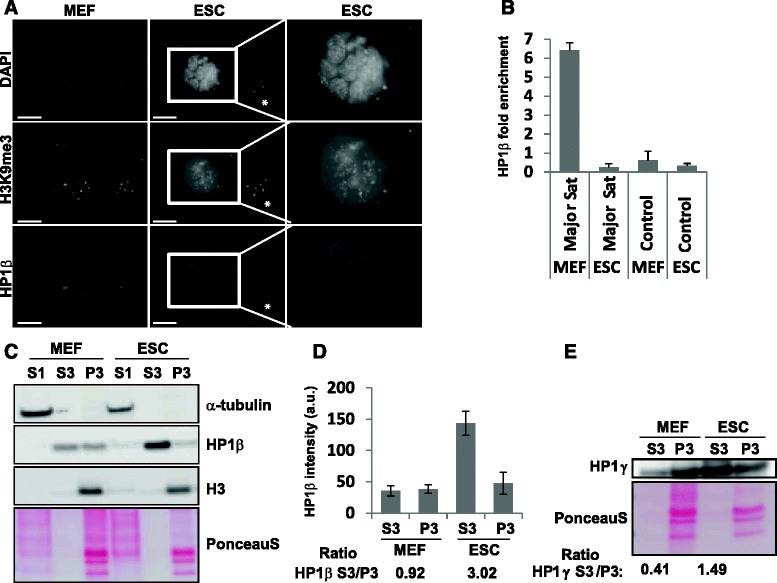
HP1β does not associate predominantly with chromatin in pluripotent cells. **a** No colocalization of HP1β with pericentromeric H3K9me3 foci in pluripotent cells. MEFs (*left*) and R1 ESCs (*middle*) were co-immunostained for DAPI (*top*), H3K9me3 (*middle*) and HP1β (*bottom*). *Right panel*: an enlargement of the ESC colony shown in the box. *Asterisks* mark examples of feeder layer MEFs used for the culture of pluripotent cells. Scale bars = 25 μm. **b** Chromatin immunoprecipitation (ChIP)-quantitative PCR for HP1β on major satellite repeats in MEFs and ESCs. HP1β is not enriched on major satellite repeats in pluripotent cells. The SSC144 region was used as control. Error bars represent standard error of the mean. **c** HP1β is predominantly nucleoplasmic/chromatin-unbound in pluripotent cells. Western blots for α-tubulin, HP1β and histone H3 in MEFs (*left*) and R1 ESCs (*right*), fractionated to the cytoplasmic fraction (*S1*), nucleoplasmic (nuclear chromatin-unbound) fraction (*S3*), and chromatin-bound fraction (*P3*). PonceauS protein staining in the histone range of the blot was used as a loading control (*bottom*). **d** HP1β levels in each fraction were quantified from three independent experiments. Error bars represent standard error of the mean; *a.u.* arbitrary units. The ratio of the nucleoplasmic fraction to the chromatin-bound fraction is indicated below for MEFs and ESCs. **e** Western blots for HP1γ in MEFs and R1 ESCs, fractionated to the cytoplasmic fraction (*S1*), nucleoplasmic (nuclear chromatin-unbound) fraction (*S3*), and chromatin-bound fraction (*P3*). Protein staining with PonceauS in the histone range of the blot was used as a loading control. The ratio of the nucleoplasmic fraction to the chromatin-bound fraction is indicated below for both MEFs and ESCs

We next asked whether the more diffuse distribution of HP1β found in ESCs versus differentiated cells reflects a different mode of binding to chromatin. To that end, we first co-stained MEFs and ESCs with the heterochromatin markers H3K9me3 and HP1β. Whereas HP1β almost completely overlapped with H3K9me3 in MEFs, consistent with recognition of H3K9me3 by its chromodomain, it did not co-localize with bright H3K9me3 foci in ESCs (Fig. 7a). In the case of HP1α, a major overlap with the H3K9me3 foci was scored in both ESCs and differentiated cells [6]. Therefore, we suggest that the correlation of HP1β with H3K9me3 by ChIP-Seq in ESCs probably does not represent HP1β association with H3K9me3-containing chromocenters, but rather recognition of this modification at other loci. On the other hand, in the somewhat less pluripotent E14 ESCs, the few HP1β foci that we observed did co-localize largely with H3K9me3 heterochromatin (Figure S6c in Additional file 6).

We next performed ChIP-qPCR to test the association of HP1β with major satellite repeats in ESCs. The major satellite is the main sequence element in heterochromatic pericentromeric regions and these generally map to the chromocenters where HP1β binds in differentiated cells [53, 62]. Unlike the situation in MEFs, HP1β was not highly enriched on major satellite repeats in pluripotent ESCs (Fig. 7b). These results are consistent with a recent study in which HP1β was shown to be only moderately enriched at pericentromeric regions in ESCs, while HP1α was strongly enriched at these sites, as monitored by a quantitative locus purification method [63]. The large absence of HP1β on major satellites in pluripotent ESCs compared with MEFs is consistent and reinforces the almost complete absence of pericentromeric foci enriched with HP1β in ESCs.

In order to measure the association of HP1β with chromatin in differentiated and undifferentiated cells biochemically, we fractionated MEFs and ESCs into cytoplasmic (S1), nucleoplasmic/chromatin unbound (S3) and chromatin-bound (P3) fractions, and analyzed HP1β levels in each fraction using immunoblots. Interestingly, HP1β was highly enriched in the nucleoplasmic fraction of ESCs, and was only weakly associated with the chromatin fraction, whereas in the differentiated MEFs, HP1β was more enriched in the chromatin-bound fraction (Fig. 7c, d).

We obtained similar results for HP1γ (Fig. 7e), which also displayed a diffuse nuclear localization in pluripotent ESCs (Fig. 5d, e). This is in contrast to HP1α distribution, which largely overlaps with pericentromeric heterochromatic foci at all stages of differentiation (data not shown and [6]). Finally, to test whether HP1β and HP1γ have redundant functions in ESCs, we knocked down over 70 % of the level of HP1γ by small interfering RNA (siRNA) in the HP1β^−/−^ ESCs (Figure S6d in Additional file 6), and found that depletion of HP1γ led to a slight (~18 %, *p* = 0.01) reduction in the proliferation rate of WT cells (Figure S6e in Additional file 6) [15, 51], yet there were no additive effects on cell growth and survival in the HP1β KO/HP1γ knock-down (Figure S6f in Additional file 6).

Taken together, we conclude that, unlike the situation in differentiated cells, HP1β does not associate predominantly with chromatin in ESCs, does not localize to pericentromeric H3K9me3 foci, and is not enriched on major satellite repeats. Importantly, we show by ChIP-Seq that HP1β in ESCs is enriched on exons over the genome, even though this may represent a minor fraction of total HP1β in ESCs, given that most HP1β is not chromatin-bound. The distribution and expression levels of HP1β and HP1γ are similar, yet loss of HP1β in ESCs resulted in precocious differentiation in cultured ESCs, and HP1β^−/−^ embryos died perinatally [34], while depletion of HP1γ affected cell growth and differentiation only under certain conditions [51]. Thus, this dual and opposing function in pluripotent and differentiated cells appears to be unique to HP1β and is not shared redundantly with HP1γ or HP1α.

Here we have reported unique characteristics and an unexpected role for HP1β in mouse ESCs. Functionally, we found that HP1β is required to maintain the undifferentiated/pluripotent ESC state, given that HP1β depletion in both ESCs and iPSCs resulted in precocious differentiation. The differentiation was mostly towards neuronal cell types. This is in line with the aberrant cerebral cortex development phenotype observed in vivo in the HP1β^−/−^ mutant mice [34], which die around birth with defective cerebral corticogenesis and reduced proliferation of neuronal precursors. Whereas HP1β^−/−^ MEFs proliferate at a similar rate to that of WT MEFs, HP1β^−/−^ ESCs display slower proliferation rates than WT or HP1α^−/−^ ESCs, in conjunction with other observations [64].

A meta-analysis of all available ChIP-Seq datasets in ESCs [65] revealed that the HP1β promoter is bound by Oct4, Nanog, Klf4, Esrrb, Nr5a2, and Sall4, which are all factors of the pluripotency network. This may well account for the high levels of HP1β in ESCs. Indeed, a knockdown of Oct4 in ESCs downregulated HP1β, while knockdown of Nanog or Klf4 did not [66]. However, we have made the intriguing finding that the depletion of HP1β in ESCs leads to the downregulation of most of the key pluripotency factors, including Nanog, Klf4, and Esrrb, but not of Oct4 (Fig. 5d). This suggests that Oct4 acts upstream of HP1β, and may be responsible for the high expression level of HP1β in ESCs. This in turn appears to contribute by regulating the other pluripotency factors. Nonetheless, the effect of HP1β on the global pluripotency gene expression signature does not appear to be through direct transcriptional control. One possible mode of action is that the nucleoplasmic fraction of HP1β stabilizes or potentiates selected long intergenic non-coding RNAs (lincRNAs) that were shown to associate with HP1β in ESCs and to regulate pluripotency [67]. While this is possible, further studies are needed to examine the effects of HP1β loss on lincRNAs in ESCs and the role of potential HP1β–RNA complexes on pluripotency.

The diffuse localization of HP1β in undifferentiated ESCs remains particularly intriguing, especially since H3K9me3 and HP1α foci are clearly visible [2, 13]. This rules out the possibility that the diffuse localization of HP1β is due to the absence of pericentromeric foci in ESCs, and suggests that HP1β has a differential affinity for H3K9me3 in ESCs versus differentiated cells [68]. This may reflect the preferential binding of HP1β to another histone modification that prevents or competes for its binding to H3K9me3, or else, possibly, competition for HP1β between RNA and H3K9me3-containing nucleosomes. We can rule out a role for H3S10 phosphorylation in this phenomenon, as we see no differences in H3S10P in ESCs and MEFs (data not shown). We do not rule out, however, that other histone modifications that are differentially abundant in pluripotent and differentiated cells might impact HP1β localization [69–71]. HP1β in vivo undergoes multiple post-translational modifications, including acetylation, phosphorylation, methylation, and many more [72], and several of these modifications have been correlated with the different functions of HP1 [21, 73–75]. Thus, HP1β itself could be differentially modified in pluripotent and differentiated cells. Alternatively, in order to explain HP1β diffuse localization in ESCs, HP1β may be targeted to sites of action by binding differentially to KAP1/TRIM28/TIF1β [76] in pluripotent versus differentiated cells, although this interaction was not detected under our LC-MS/MS experimental conditions.

Our findings suggest that HP1β has distinct interaction partners in ESCs compared with differentiated MEFs. In MEFs, HP1β interacting partners could be classified into the following categories: ‘cell structure and motility’, including actin, myosin, lamin, and other filaments; ‘protein biosynthesis’, including mostly ribosomal proteins; ‘chromatin and nucleotide’; and ‘RNA processing’ (Additional file 4). Based on these findings, we speculate that HP1β association with nuclear filaments such as lamin, myosin, and/or tubulin may contribute to its association with stable heterochromatic foci in differentiated cells (MEFs). The interaction of HP1β with an RNA-processing protein category also led us to wonder whether this category of proteins could be involved in the silencing function of HP1β in differentiated cells. In addition to the conventional mechanism of transcriptional repression by heterochromatin, we propose that HP1β and RNA-processing proteins could serve to recognize RNA transcribed from heterochromatin, leading to its sequestration and/or degradation. Such a role has been reported for the HP1^Swi6^ protein in fission yeast [77]. In addition, association between *Drosophila* HP1a and a broad set of repetitive RNAs has been recently reported [78], and interactions between HP1a, RNA transcripts, and some RNA-processing heterogeneous nuclear ribonucleoproteins (hnRNPs) were also shown to be involved in regulation of gene expression and heterochromatin formation [79].

## Conclusions

We propose that HP1β has two distinct roles in chromatin modulation that depend on the differentiation state of the cell. Functionally, we found that HP1β is required to maintain the undifferentiated/pluripotent ESC state, whereas differentiated cells, such as EBs or MEFs, fail to maintain a proper differentiation state in the absence of HP1β, and reprogram into iPSCs more easily than WT cells. These distinct functional roles of HP1β are manifest in our findings that HP1β has different protein levels, nuclear distributions, binding sites on chromatin, and protein binding partners at different stages of differentiation. Future work will define the mode of action with respect to the maintenance of pluripotency as well as the role of HP1β in stabilizing differentiated states.

## Materials and methods

### Cells and cell culture

Mouse ESCs, including R1, E14, HP1α^−/−^, HP1β^−/−^, their WT littermate control line HM1 [52], iPSC lines Rr5 [14], and WT, HP1α^−/−^, and HP1β^−/−^ iPSCs (this study) were cultured in 5 % CO_2_ at 37 °C on gelatin-coated dishes and mitomycin-C treated MEF feeder layer in standard ESC media Dulbecco’s modified Eagle medium (DMEM) containing 10 % ESC-qualified fetal calf serum (FCS), 1000 U/ml LIF, 0.1 mM nonessential amino acids, 1 mM sodium pyruvate, 2 mM L-glutamine, 50 μg/ml penicillin-streptomycin, 100 μM β-mercaptoethanol). All cell culture reagents were purchased from GIBCO-BRL (Invitrogen, Carlsbad, CA, USA). Primary MEFs, derived from embryos at E13.5, were grown in DMEM containing 10 % FCS, 2 mM L-glutamine and 50 μg/ml penicillin-streptomycin. MEFs (WT, HP1α^−/−^, HP1β^−/−^) which were used to generated iPSCs were derived from embryos of the corresponding genotypes [34, 52].

### Immunofluorescence and antibodies

Cells were plated on round sterilized 12 mm coverslips in 24-well culture plates (Greiner), coated with gelatin and pre-plated with mitomycin-C treated MEFs. Cells were fixed in 4 % paraformaldehyde for 15 min at room temperature, washed twice with phosphate-buffered saline (PBS) and permeabilized with 0.5 % Triton-X for 5 min at room temperature, washed three times with PBS, and blocked for 30 min with 10 % FCS in PBS at room temperature. Primary antibodies (overnight at 4 °C) included Nanog (R&D, AF2729; 1:20), HP1α (Euromedex, 2HP-1H5-As; 1:750), HP1β (Euromedex, 1MOD-1A9; 1:1750), HP1γ (Euromedex, 2MOD-1G6; 1:750) and H3K9me3 (rabbit polyclonal kindly provided by T. Jenuwein; 1:100). Detection was with anti-rabbit or anti-mouse conjugated to Cy3 or anti-Donkey-FITC (Jackson ImmunoResearch). Images were taken at 60× with an oil NA1.4 lens using a spinning disk confocal microscope (CSUX, Yokogawa, Japan) equipped with an iXon + DU-897-BV monochrome EMCCD camera (Andor, UK) mounted on an Olympus IX81 fully automated microscope, or with an Olympus IX71 epifluorescent microscope equipped with a Dp71 camera (Olympus).

### Image analysis

Image analysis was performed as previously described [14]. Fluorescence intensity was measured in confocal sections where each nucleus was at its optimal focal plane and clearly distinguishable from surrounding nuclei in the Z-stack using ImageJ [80]. Intensity and nuclear size were measured in a semi-automated manner. Heterochromatin foci were also measured in a semi-automatic manner using the ‘analyze particles function’ in the ImageJ software.

### ESC differentiation

For EBs, R1 ESCs were cultured in suspension in Petri dishes in standard ESC media without LIF. For NPC differentiation, ESCs were separated from MEFs and grown in suspension on Petri dishes without LIF for 4 days to allow for EB formation. EBs were replated on polylornithine/fibronectin (Sigma) coated eight-well μ-Slides (ibidi, Munich, Germany) in DMEM/F12 medium supplemented with ITS (5 mg/ml insulin, 50 mg/ml transferrin, 30 nM selenium chloride) and fibronectin (5 mg/ml) and grown for 2–6 days longer until NPC day 6–10, respectively. The antibodies used to immunostain NPCs included anti-Tuj1 (MAB1637, 1:200) and anti-Nestin (#130, 1:100), a generous gift from Dr. Ron McKay.

### Teratoma formation

Teratomas were produced as previously described [14]. Briefly, 10^6^ ESCs were suspended in 35 μl ESC medium and 15 μl Matrigel™ (BD Biosciences). This mix was injected subcutaneously into the dorsal flank of SCID mice (C.B-17/lcrHsd-SCID-bg). Three weeks after the injection, teratomas were surgically dissected. Samples were weighed, fixed in PBS containing 4 % paraformaldehyde, and embedded in paraffin. Sections were stained with hematoxylin and eosin. The joint ethics committee (IACUC) of the Hebrew University and Hadassah Medical Center approved the study protocol for animal welfare. The Hebrew University is an AAALAC International accredited institute. All animal experiments were conducted in accordance with the Hebrew University’s animal committee, ethical approval number IACUC:NS-09-11616-4.

### Cell fractionation and immunoblots

Protein fractionation in the chromatin-bound, the nucleoplasmic or the cytoplasmic compartments was performed essentially as described [81]. The protein extraction was performed on 4 × 10^7^ primary MEFs (passage 3) or on 4 × 10^7^ R1 mouse ESCs. The protein fractions were separated on 4–20 % gradient Bis-Tris SDS gels (BioRad), blotted, and incubated with the following primary antibodies: HP1β (1MOD-1A9, Euromedex; 1:2000), HP1γ (2MOD-1G6, Euromedex; 1:2000), H3K9me3 (rabbit polyclonal; 1:100), kindly provided by T. Jenuwein (Freiburg), histone H3, kindly provided by M. Bustin (1:10,000, rabbit), and alpha tubulin (#ACLX135B, Accurate Chemical & Scientific Corporation). Other antibodies used for western blots included lamin A/C (sc-20680, SantaCruz; 1:100), hnRNPa2/b1 (ab31645, Abcam; 1:200), phosphoserine (ab9332, Abcam; 1:100) and phosphothreonine (Cell Signaling #93865; 1:3000).

### Cell proliferation assay

ESCs were plated at similar conditions and passage number at a density of 2.5 × 10^5^ cells per 10 cm plate, and counted in triplicates after 24, 48 and 96 h of culture. The cell proliferation assay shown in Additional file 8 was performed similarly but in 12-well plates with 10^5^ cells initially plated.

### Magnetic cell separation based on SSEA1 expression

Magnetic separation was done according to Miltenyi Biotec instructions using anti-SSEA-1 (CD15) microbeads (130-094-530). We confirmed a homogeneous cell population by obtaining small HP1β^−/−^ ESC colonies following plating of the sorted SSEA-1-positive cells.

### Microarrays and data analysis

Microarray analysis was performed with Affymetrix Exon Arrays *MoEx-1_0-st-v1*, with RNA purified from ESCs and EBs using the RNeasy Mini Kit (Qiagen) supplemented with DNaseI. Two biological samples of each cell type were analyzed: ES HM1 WT, ES HP1α KO or ES HP1β KO, and the derived EBs HM1 WT, EBs HP1α KO, or EBs HP1β KO. Quality and the comparability of the datasets were verified with the Affymetrix Expression Console software. Datasets in duplicate were compared with their WT counterparts and only genes that were either upregulated or downregulated in pairwise comparisons were selected for further analyses. A gene was considered differentially expressed only if the detected signal was above the background (>45) in at least one of the compared samples. The expression data files are available from the Gene Expression Omnibus (GEO) database [GEO:GSE65121].

### Reverse transcription-PCR

Total RNA was purified using RNeasy Mini Kit (Qiagen). Two micrograms of RNA treated with DNase I (Qiagen) were reverse-transcribed using a high capacity reverse transcriptase kit (Applied Biosystems) according to the manufacturer’s instructions. qRT-PCR was done with Power SYBR® Green PCR Master Mix (Applied Biosystems) using the Bio-Rad CFX96 real-time system and the following primer sets (Fwd = forward, Rev = reverse):

- Grb10: Fwd-TGCCGAAGATGAGCAGATCCGT, Rev-CACTGCGCATAGGTGCGTTGA
- Bmp4: Fwd-CCAGTCTCTGGCCCTCGACC, Rev-GGAATGGCTCCATTGGTTCCTGC
- Mylpf: Fwd-AGCGGAAGGGAGCTCCAACG, Rev-AGACGGCCCATGGCTGCAAA
- Car4: Fwd-TGGGCAGCGTCTTTCCCCTC, Rev-ACTTCTCAGGCCCCAAGCAACT
- Ff15: Fwd-TGTGGACTGCGAGGAGGACCA, Rev-CCGAGTAGCGAATCAGCCCGTA
- Suv39h1: Fwd-GCGACTACCCCGCATCGCAT, Rev-GTCCACGGGGTCCACTTGCAT
- Nanog: Fwd-AGGGTCTGCTACTGAGATGCT, Rev-CAACACCTGGTTTTTCTGCCACCG
- Nestin: Fwd-TCAGATCGCTCAGATCCTGGA, Rev-GGTGTCTGCAAGCGAGAGTTCT
- Klf4: Fwd-TGGTAAGGTTTCTCGCCTGT, Rev-CCTGTGTGTTTGCGGTAGTG
- Cbx3: Fwd-GGTCCAGGTCAGCCAGTCTA,Rev-CCAGCCACGATTCTATTTCC
- GAPDH: Fwd-GTGTTCCTACCCCCAATGTGT, Rev-ATTGTCATACCAGGAAATGAGCTT

Data were normalized to GAPDH control. Agreement between the fold changes found in qRT-PCR and in the microarray analysis was calculated as r^2^ using the trendline option in Excel.

### ChIP-qPCR for macro-satellites

ChIP was performed as previously described with a few modifications [82, 83]. Briefly, chromatin solution from R1 ESCs and MEFs was pre-cleared with a protein G-agarose 50 % gel slurry (SC-2002) for 45 min at 4 °C and immunoprecipitated overnight at 4 °C using the following antibodies: mouse anti-HP1β (Euromodex-1 MOD-1A9-AS; 2 μg), and the control mouse anti-IgG (Sigma I5381; 2 μg). Real-time PCR (Applied Biosystems) reactions were performed in triplicate. The primers used in order to assess the transcriptional level of the major satellites are described in [84], and the control primers used were: Slc44a1 Fwd- TCTGTCAGTCCGTGAATGGTGGTT, Rev- ACCACTTCCTTCGTGGAAAGGACA.

### Co-immunoprecipitation

The nuclear (S3 and P3) fractions of 10^8^ MEF or R1 ESCs were used as extracts for immunoprecipitation. Immunoprecipitations were done using antibodies for HP1β (1MOD-1A9, Euromedex) and GFP (#11814460001, Roche; negative control). Protein G-Agarose beads (Roche Applied Science) were washed extensively with wash buffer (30 mM Tris–HCl pH 7.5, 0.2 mM EDTA, 0.5 mM dithiothreitol, 0.2 % Triton X-100, 150 mM NaCl), centrifuged on a 30 % sucrose cushion and washed with 30 mM Tris pH 7.5. The bound proteins were subjected in part to SDS-PAGE silver staining and western blots and analyzed using LC-MS/MS.

### LC-MS/MS

Gel pieces were de-stained and proteins were reduced by dithiothreitol (DTT) and alkylated by iodoacetamide (IAA). Tryptic digestion was performed using porcine trypsin (Sequencing Grade Modified, Promega, WI, USA) overnight. The tryptic peptides were extracted by 5 % formic acid in 50 % acetonitrile and vacuum dried by speedvac. Each dried fraction was reconstituted in 10 μl of 0.1 % formic acid and analyzed on a Dionex Ultimate 3000 RSLCnano system coupled to a LTQ-FT Ultra mass spectrometer (Thermo Electron, Bremen, Germany). The peptide separation was performed in a capillary column (75 μm inner diameter × 15 cm) packed with C18 AQ (5 μm particles, 300 Å pore size; Michrom Bioresources, Auburn, CA, USA). Mobile phase A (0.1 % formic acid in water) and mobile phase B (0.1 % formic acid in acetonitrile) were used to establish a 90-min gradient comprising 3 min of 0–5 % B and then 52 min of 5–25 % B followed by 19 min of 25–80 % B, maintenance at 80 % B for 8 min, and finally re-equilibration at 5 % B for 8 min. The HPLC system was operated at a constant flow rate of 300 nL/min. The sample was injected into an LTQ-FT through an ADVANCE CaptiveSpray source (Michrom Bioresources) with an electrospray potential of 1.5 kV. The gas flow was set at 2, ion transfer tube temperature was 180 °C, and collision gas pressure was 0.85 millitorr. The LTQ-FT was set to perform data acquisition in the positive ion mode as described previously [85]. Proteins were identified by Mascot search against the IPI_mouse database, as described previously [85]. The list of significant protein hits from the co-immunoprecipitation samples was compared with the negative control samples. Proteins found in control samples were excluded.

### Reprogramming experiments

Reprogramming was conducted with a third generation lenti-vector EF1α-STEMCCA that expresses a single multicistronic transcript of the four factors (Oct4, Klf4, Sox2, and c-Myc) [86]. 293-T cells in a 14-cm culture dish of 70 % confluency were transfected with 5-plasmid system using Trans-IT transfection reagent (Mirus). Primary MEFs at passage 4 were seeded at 2 × 10^5^ cells per 10 cm dish. Virus-containing supernatants derived from the 293-T culture 48 and 72 h after transduction were filtered through a 0.45 μm cellulose acetate filter and supplemented with 4 μg/ml polybrene. Each culture of primary MEFs had two rounds of overnight infection with virus/polybrene-containing supernatants diluted 1:10 in MEF medium. After infection, the cells were washed with PBS and MEF medium for 2 days. On day 2, cells were re-plated on gelatin coated six-well plates on top of mitomycin-C-treated MEFs in standard ESC media. iPSC clones were selected according to their morphology on day 12.

### Knockdown experiments

For HP1γ knockdown, 10^5^ cells per well were seeded in 12-well plates on a feeder layer of MEFs. Three different conditions were used in the assay: no treatment, siControl (Dharmacon, ON-TARGETplus Non-targeting Control Pool), and siHP1γ (ON-TARGET plus Mouse Cbx3 siRNA SMARTpool: L-044218-01-0005). siRNAs were transfected at 50 nM final concentration using lipofectamine 2000 (Life Technologies). Cells were counted every 24 h using an automated cell counter (TC10, Bio-Rad). HP1β knockdown in R1 ESCs was performed using siGENOME siRNAs (Dharmacon) with Lipofectamine 2000 (Invitrogen) according to the manufacturer’s instructions along with a scrambled control. Cells were grown for an additional 48 h before cell fixation.

### ChIP-Seq

ChIP-Seq experiments were performed on E14 ESCs from 129P2/Ola mice [87]. For each sample, 10^6^ cells were crosslinked with 1 % formaldehyde and cell nuclei were prepared using swelling buffer (25 mM HEPES, pH 7.8, 1 mM MgCl_2_, 10 mM KCl, 0.1 % NP-40, 1 mM DTT). Chromatin was sheared to 220 bp fragments. After IgG preclearance the sheared chromatin was incubated with 4 μg of HP1β (Euromedex, 1MOD-1A9-AS) antibody overnight. After washes with sonication buffer (10 mM Tris–HCl, pH 8.0, 200 mM NaCl, 1 mM EDTA, 0.5 % N-lauroylsarcosine, 0.1 % Na-deoxycholate), high-salt-buffer (50 mM HEPES pH 7.9, 500 mM NaCl, 1 mM EDTA, 1 % Triton X-100, 0.1 % Na-deoxycholate, 0.1 % SDS), lithium buffer (20 mM Tris–HCl pH 8.0, 1 mM EDTA, 250 mM LiCl, 0.5 % NP-40, 0.5 % Na-deoxycholate) and 10 mM Tris–HCl, chromatin was eluted from the protein G magnetic beads and the crosslink was reversed overnight. After RNase A and proteinase K digestion, DNA was purified and cloned in a barcoded sequencing library for the Illumina HiSeq2000 sequencing platform (single reads of 50 bp length).

### ChIP-Seq data analysis

Data analysis was performed as previously described [65]. HP1β Chip-Seq reads were aligned to the mouse genome (mm9) using Bowtie [88], taking only uniquely aligned reads with no more than one mismatch. Peaks were extracted using MACS 1.4 [89], setting a minimal *p* value cutoff of 10^−3^ and a fold change range for a model building between 8 and 30. HP1β was considered to be associated with a gene if the peak was within the gene body or was considered to be associated with a promoter region if the peak was within 5 kb upstream of the transcription start site. In order to assess the correlation of HP1β with other proteins, the genome was binned into non-intersecting intervals of 3000 bases long. We next checked if peaks for a given protein can be found within the same bin as the HP1β peaks by extracting the hyper geometric *p* value (Bonferroni corrected).

### Data availability

The ChIP-Seq and microarray data are available from the GEO database (accession number [GEO:GSE65122], which groups our ChIP-Seq [GEO:GSE64946] and expression [GEO:GSE65121] data). Microscopic original data are available from the Dryad Digital Repository [90].

## Additional files

Additional file 1: Figure S1. Validation of knockout cells. **a** Immunostaining with HP1α and HP1β antibodies on WT, HP1α KO, and HP1β KO ESC colonies surrounded by MEFs (examples are marked by *asterisks*) as feeder layer. ESC colonies not easily detectable are marked with a *dashed line*. Scale bars = 25 μm. **b** Western blots for HP1β in WT and HP1β KO ESCs and EBs. **c** Co-staining with H3K9me3 antibody and DAPI in WT, HP1α KO, and HP1β KO ESC colonies surrounded by MEFs as a feeder layer. ESC colonies are marked with a *dashed line*. Scale bars = 14 μm. The DAPI staining and H3K9me3 foci allow visualization of the global DNA organization and chromocenter organization. **d** Fluorescence recovery after photobleaching (FRAP) analysis of histone H1 fused to GFP in WT and HP1β KO ESCs (n = 10). (PDF 5.30 mb) Additional file 2: Figure S2. Mitosis in WT, HP1α KO, and HP1β KO ESCs. **a** Confocal images of single mitotic nucleus of WT ESCs (*top*), HP1α KO ESCs (*middle*), and HP1β KO ESCs (*bottom*) in metaphase or anaphase immunostained for H3K9me3 (*red*) and counterstained with DAPI (*blue*). The merged images shown on the *left* allow visualization of the DNA and H3K9me3 distribution during metaphase and chromosome segregation in anaphase. Scale bars = 7 μm. **b** HP1β knockdown experiment (siRNA) in R1 ESCs. Cells were treated with control siRNA or HP1β siRNA and grown for an additional 48 h before cell fixation and immunostaining with HP1β, Nestin, and DAPI. (PDF 2.41 mb) Additional file 3: Figure S3. Microarray validation. **a** The distribution of the frequency of the fold change of the expression of genes from WT versus HP1β KO ESCs. The curve represents the cumulative percentage, which enables determination of the fold changes which are statistically significant. *P* values corresponding to 0.05 and 0.005 are shown (*light squares*). **b** Expression levels, measured by RT-qPCR, of nine representative genes shown next to the corresponding microarray results for HP1α KO (*white*) and HP1β KO (*left*) ESCs. The linear regression and correlation were calculated between the two data sets (*r* = 0.8). **c** Same as in (**b**) with the EB samples derived from WT, HP1α KO, and HP1β KO EBs. (PDF 152 kb) Additional file 4: Table S1. HP1β interacting partners in ESCs and MEFs by co-immunoprecipitation followed by LC-MS/MS analysis. Only proteins that were not identified in the corresponding control co-immunoprecipitation samples were regarded as specific. Hits found in both biological duplicates are in *bold* and *underlined* and are shown at the *top*. The eight proteins that were identified in both cell types are in *bold* and *underlined* and are shown at the *bottom*. Hits found also in negative control experiments similar to our experimental settings in the Contaminant Repository for Affinity Purification through its web interface [91] are most probably false negative interacting partners, and thus were excluded from the final list (Fig. 4a). Those few additional excluded hits are marked by an *asterisk* in the lists and are in *gray* and *italics*. (Control sets: CC76 CC78 CC79 CC80 CC81 CC82 with the following filters: Cell type-HEK293, Affitnity Support-Agaraose beads, Instrument Type for Mass Spectometry- LTQ-FT.) The HP1β interacting partners in MEFs were classified according to their functional annotation and biological process using Gene Ontology (GO). Only the different categories found to be statistically significant are indicated by colors and found in the legend of the pie chart on the right side of the excel sheet “HP1beta interactors MEF”. (XLSX 27.1 kb) Additional file 5: Figure S4. HP1β is highly expressed in ESCs. **a** Western blot for HP1β (*top panel*) and histone (*H3*, *middle panel*) in MEFs and mouse ESCs (R1). For both cell types, the two nuclear fractions that include the whole amount of HP1β and histone H3 (*S3* nucleoplasmic fraction, *P3* chromatin-bound fraction) are shown. Protein staining with PonceauS (*bottom panel*) of the blot was used as a loading control. **b** Total HP1β levels (S3 + P3) were quantified in MEFs versus ESCs from three western blot experiments and normalized to H3 levels; error bars represent standard error of the mean. **c** Western blot for HP1β in ESCs (R1) and embryoid bodies (EBs) after 7 days of differentiation. Protein staining with PonceauS in the HP1 range of the blot was used as a loading control. **d** Global view of the expression level of CBX1/HP1β in human pluripotent cells (hESCs and hIPSCs) and in differentiated cells produced by the Amazonia! tool from public human transcriptome datasets [56]. (PDF 255 kb) Additional file 6: Figure S5. HP1β is diffuse in fully pluripotent cells. **a** R1 ESC colonies surrounded by MEFs used as a feeder layer for the ESCs and as a staining positive control were co-immunostained with DAPI, Nanog, and HP1β. Images with long and short exposures are shown for the HP1β staining. Scale bars = 25 μm. **b** R1 and E14 ESCs were co-immunostained with Nanog and HP1β. The ESC colony in the *marked area* is enlarged in the *right panel*. Scale bars = 25 μm. **c** Co-immunostaining with H3K9me3 and HP1β. The R1 ESC colony (from Fig. 3) is shown on the *right* for easier comparison. Scale bars = 25 μm. **d** Relative levels of HP1γ transcripts (Cbx3) following siRNA treatment measured by RT-qPCR. **e, f** Cell proliferation assays performed in triplicate in six-well plates. The graphs show the number of WT ESCs (**e**) and HP1β KO ESCs (**f**) treated with siRNA against HP1γ or control siRNA every 24 h. (PDF 190 kb) Additional file 7: Video 1. HP1β is diffuse in nuclei of undifferentiated/pluripotent murine cells and localizes in foci in the course of differentiation. Time lapse spinning disk confocal video of ESCs expressing the endogenous HP1β fused to mCherry induced to differentiate with 1 μM of retinoic acid for 40 h (time scale in the *right upper part* of the video). (AVI 16.5 mb) Additional file 8: Figure S6. HP1β ChIP-Seq analysis. **a** Enriched categories in Gene Ontology (GO) analysis performed for the HP1β-bound genomic regions. **b** Correlation analysis of HP1β ChIP-Seq data with other existing genome-wide datasets in mouse ESCs. **c, d** Correlation analysis of the upregulated and downregulated genes in HP1β KO ESC samples compared to WT, with HP1β-bound promoters or HP1β-bound gene bodies in WT ESC samples. **e** List of downregulated and upregulated genes in HP1β KO ESC samples which are also genomically bound by HP1β in WT cells. (PDF 1.19 mb)

## Abbreviations

ChIP: chromatin immunoprecipitation
DMEM: Dulbecco’s modified Eagle medium
DTT: dithiothreitol
E: embryonic day
EB: embryoid body
ESC: embryonic stem cell
FCS: fetal calf serum
GEO: Gene Expression Omnibus
GFP: green fluorescent protein
GO: Gene Ontology
H3K: histone H3 lysine
hnRNP: heterogeneous nuclear ribonucleoprotein
HP1: Heterochromatin Protein 1
iPSC: induced pluripotent stem cell
KO: knockout
LC-MS/MS: liquid chromatography-tandem mass spectrometry
LIF: leukemia inhibitory factor
lincRNA: long intergenic non-coding RNA
MEF: mouse embryonic fibroblast
NPC: neuronal progenitor cell
PBS: phosphate-buffered saline
qRT-PCR: quantitative reverse transcription polymerase chain reaction
siRNA: small interfering RNA
WT: wild type
