# Supplementary figures and images for: Heterochromatin Protein 1β (HP1β) has distinct functions and distinct nuclear distribution in pluripotent versus differentiated cells
Source: Genome Biol. 2015 Sep 28;16:213. doi: 10.1186/s13059-015-0760-8 (PMC4587738; doi:10.1186/s13059-015-0760-8)

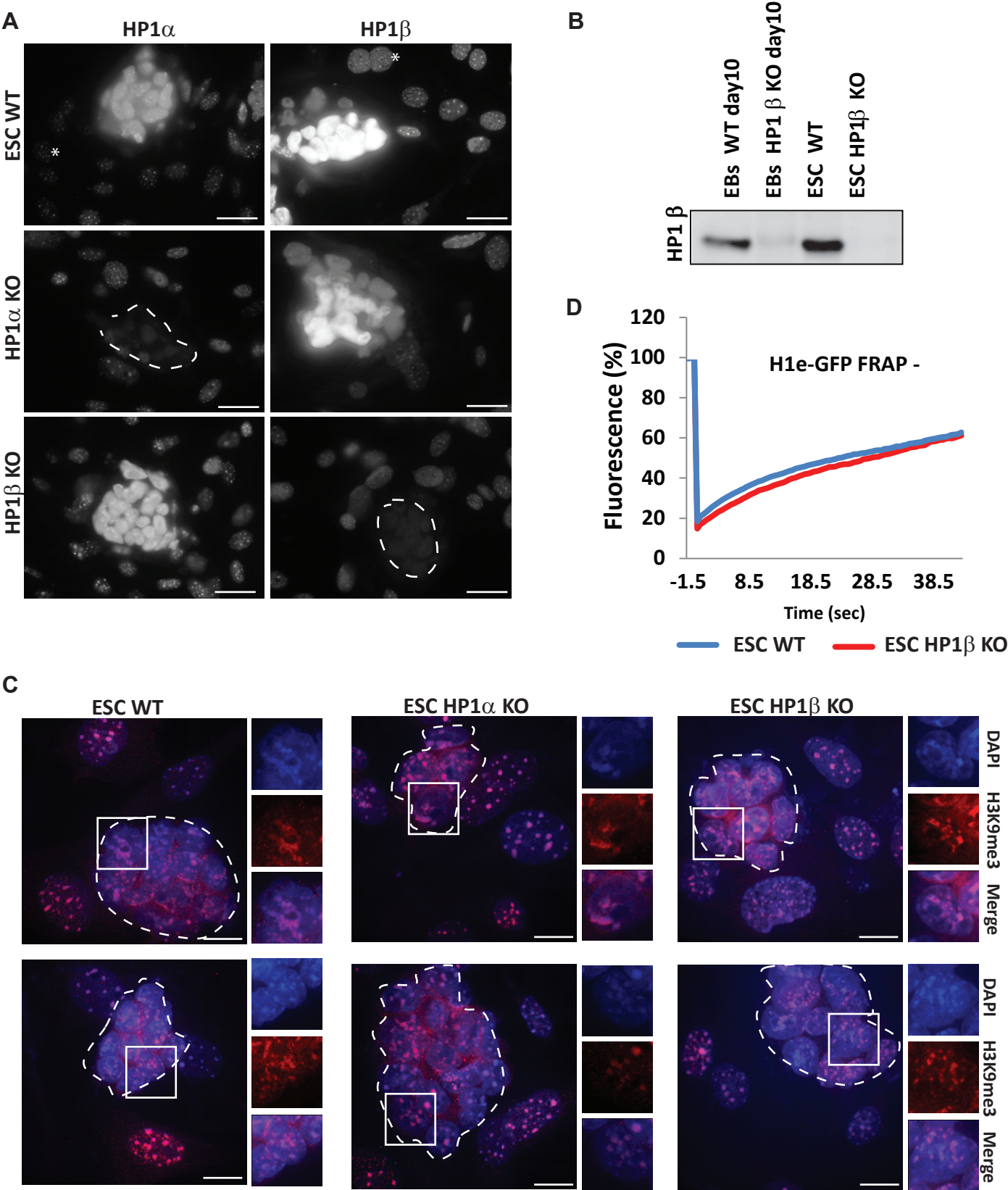

Supplement: Additional file 1: Figure S1. — Validation of knockout cells. a Immunostaining with HP1α and HP1β antibodies on WT, HP1α KO, and HP1β KO ESC colonies surrounded by MEFs (examples are marked by asterisks) as feeder layer. ESC colonies not easily detectable are marked with a dashed line. Scale bars = 25 μm. b Western blots for HP1β in WT and HP1β KO ESCs and EBs. c Co-staining with H3K9me3 antibody and DAPI in WT, HP1α KO, and HP1β KO ESC colonies surrounded by MEFs as a feeder layer. ESC colonies are marked with a dashed line. Scale bars = 14 μm. The DAPI staining and H3K9me3 foci allow visualization of the global DNA organization and chromocenter organization. d Fluorescence recovery after photobleaching (FRAP) analysis of histone H1 fused to GFP in WT and HP1β KO ESCs (n = 10). (PDF 5.30 mb) [file 13059_2015_760_MOESM1_ESM.pdf]

Additional Figure 2    Mattout et al.,

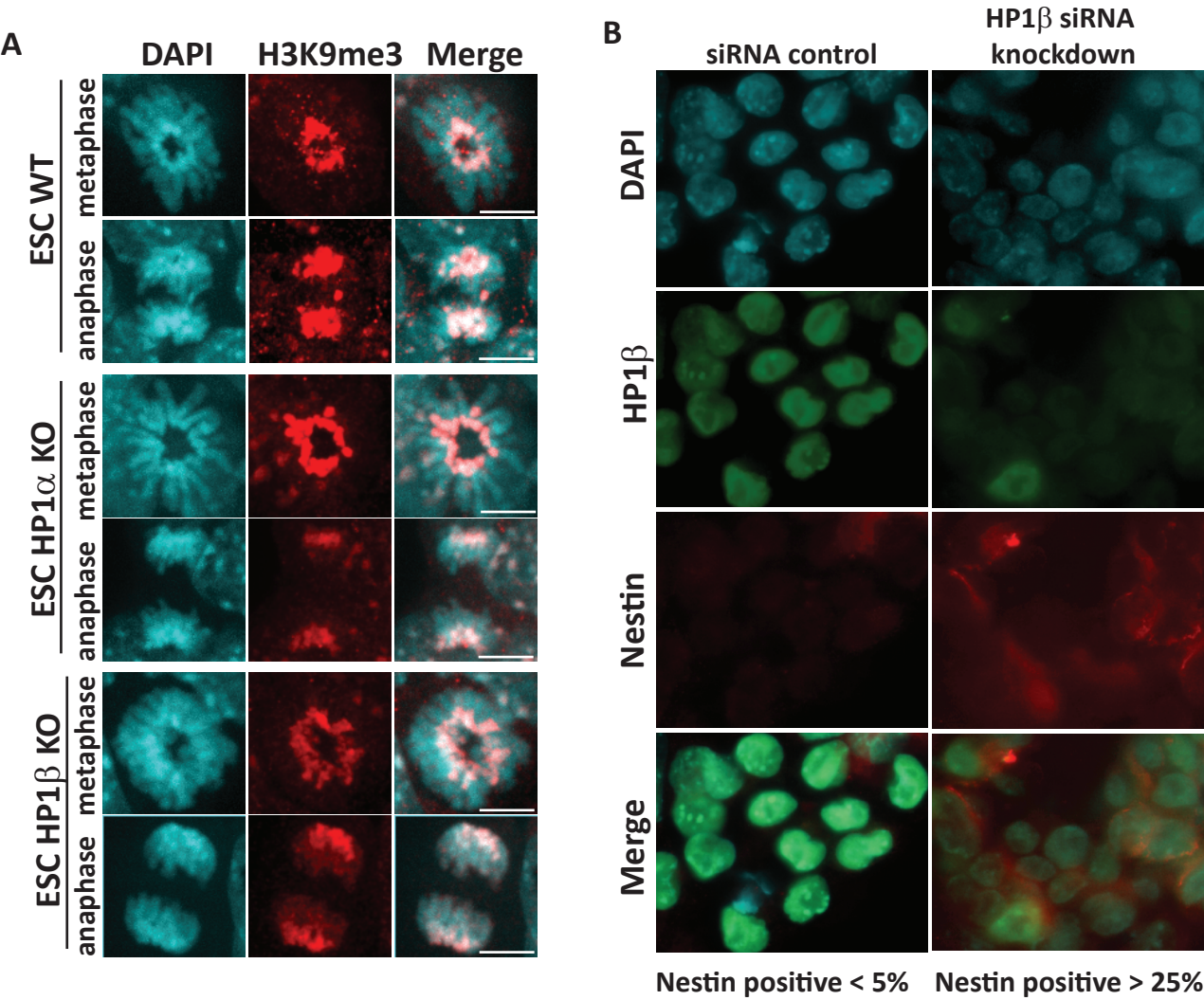

Supplement: Additional file 2: Figure S2. — Mitosis in WT, HP1α KO, and HP1β KO ESCs. a Confocal images of single mitotic nucleus of WT ESCs (top), HP1α KO ESCs (middle), and HP1β KO ESCs (bottom) in metaphase or anaphase immunostained for H3K9me3 (red) and counterstained with DAPI (blue). The merged images shown on the left allow visualization of the DNA and H3K9me3 distribution during metaphase and chromosome segregation in anaphase. Scale bars = 7 μm. b HP1β knockdown experiment (siRNA) in R1 ESCs. Cells were treated with control siRNA or HP1β siRNA and grown for an additional 48 h before cell fixation and immunostaining with HP1β, Nestin, and DAPI. (PDF 2.41 mb) [file 13059_2015_760_MOESM2_ESM.pdf]

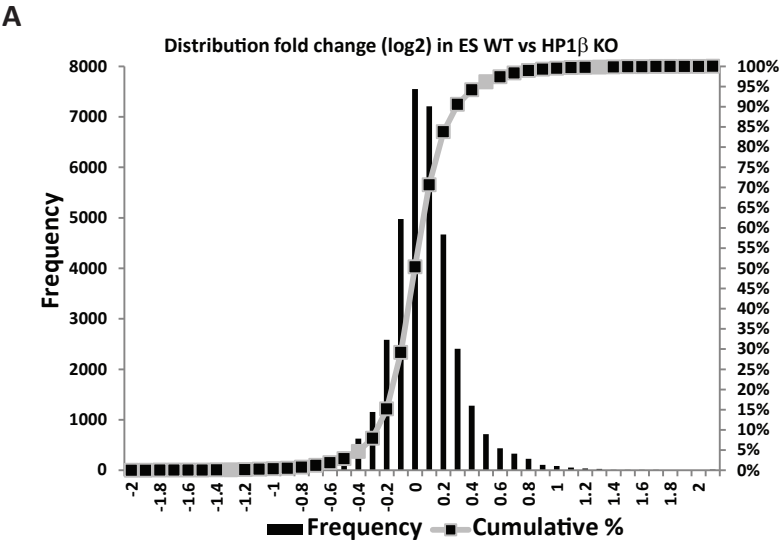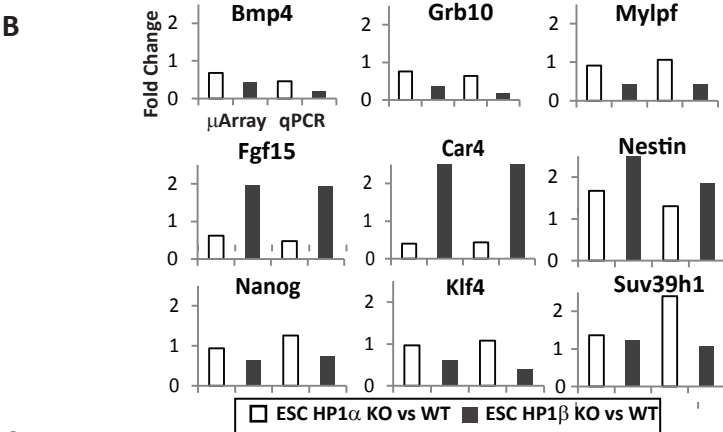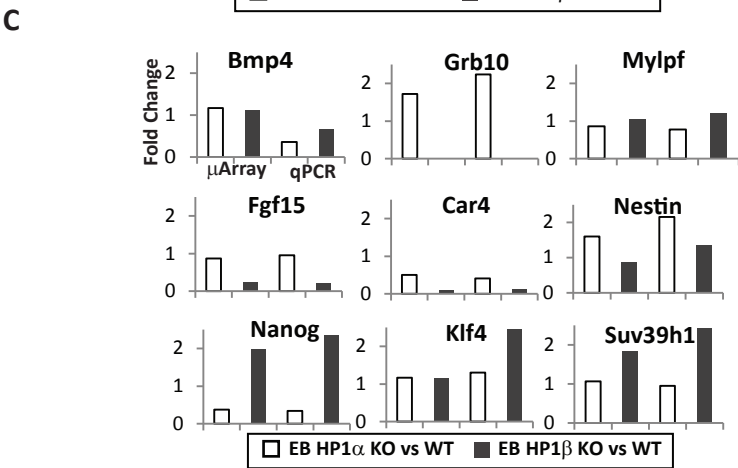

Supplement: Additional file 3: Figure S3. — Microarray validation. a The distribution of the frequency of the fold change of the expression of genes from WT versus HP1β KO ESCs. The curve represents the cumulative percentage, which enables determination of the fold changes which are statistically significant. P values corresponding to 0.05 and 0.005 are shown (light squares). b Expression levels, measured by RT-qPCR, of nine representative genes shown next to the corresponding microarray results for HP1α KO (white) and HP1β KO (left) ESCs. The linear regression and correlation were calculated between the two data sets (r = 0.8). c Same as in (b) with the EB samples derived from WT, HP1α KO, and HP1β KO EBs. (PDF 152 kb) [file 13059_2015_760_MOESM3_ESM.pdf]

Additional Figure 4

Mattout *et al.*,

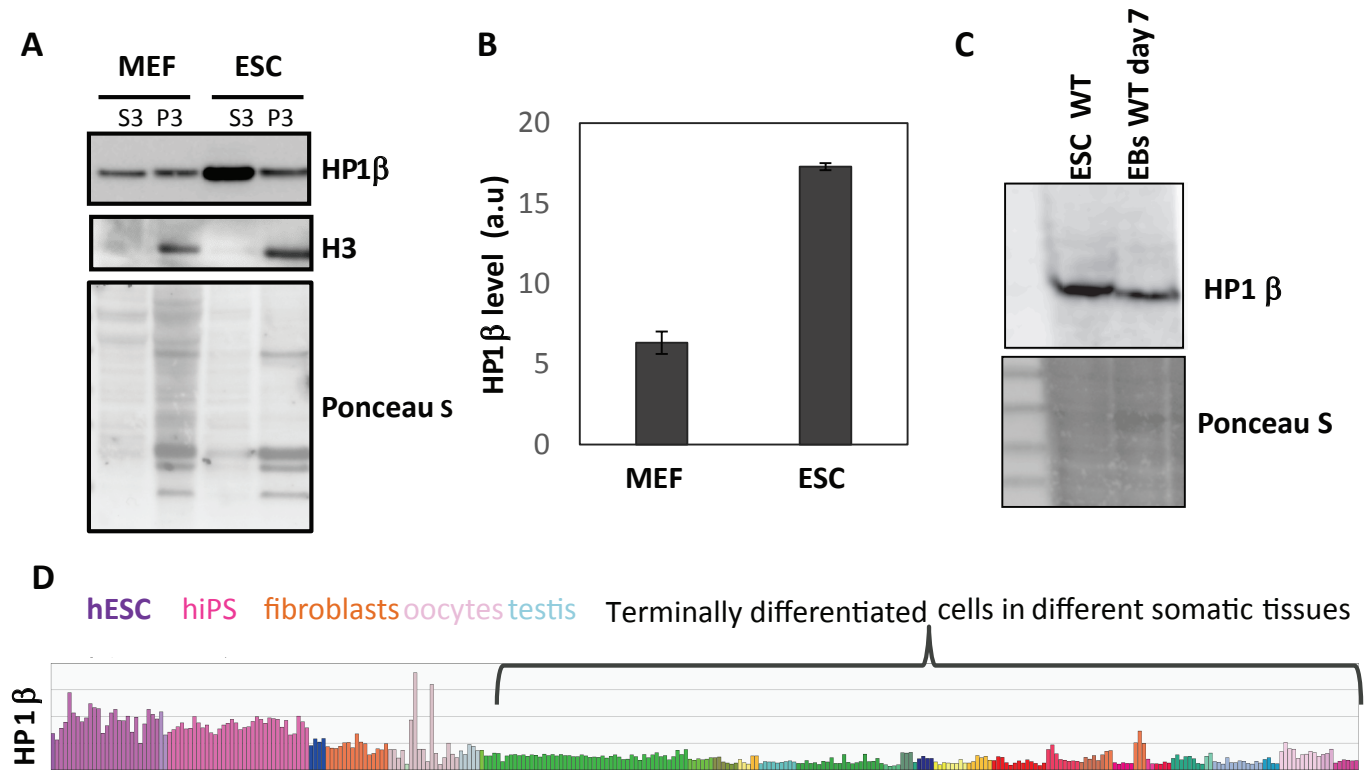

Supplement: Additional file 5: Figure S4. — HP1β is highly expressed in ESCs. a Western blot for HP1β (top panel) and histone (H3, middle panel) in MEFs and mouse ESCs (R1). For both cell types, the two nuclear fractions that include the whole amount of HP1β and histone H3 (S3 nucleoplasmic fraction, P3 chromatin-bound fraction) are shown. Protein staining with PonceauS (bottom panel) of the blot was used as a loading control. b Total HP1β levels (S3 + P3) were quantified in MEFs versus ESCs from three western blot experiments and normalized to H3 levels; error bars represent standard error of the mean. c Western blot for HP1β in ESCs (R1) and embryoid bodies (EBs) after 7 days of differentiation. Protein staining with PonceauS in the HP1 range of the blot was used as a loading control. d Global view of the expression level of CBX1/HP1β in human pluripotent cells (hESCs and hIPSCs) and in differentiated cells produced by the Amazonia! tool from public human transcriptome datasets [56]. (PDF 255 kb) [file 13059_2015_760_MOESM5_ESM.pdf]

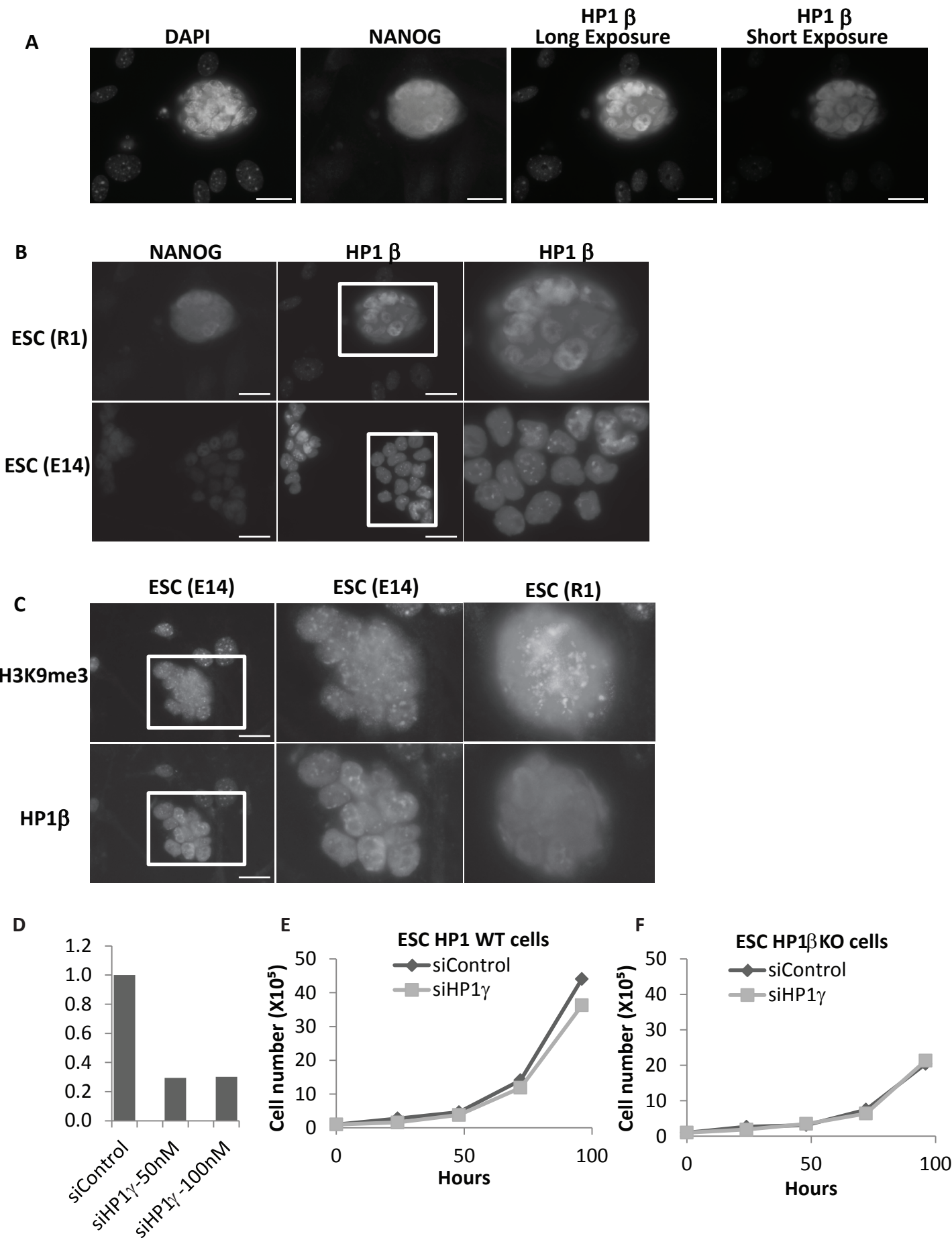

Supplement: Additional file 6: Figure S5. — HP1β is diffuse in fully pluripotent cells. a R1 ESC colonies surrounded by MEFs used as a feeder layer for the ESCs and as a staining positive control were co-immunostained with DAPI, Nanog, and HP1β. Images with long and short exposures are shown for the HP1β staining. Scale bars = 25 μm. b R1 and E14 ESCs were co-immunostained with Nanog and HP1β. The ESC colony in the marked area is enlarged in the right panel. Scale bars = 25 μm. c Co-immunostaining with H3K9me3 and HP1β. The R1 ESC colony (from Fig. 3) is shown on the right for easier comparison. Scale bars = 25 μm. d Relative levels of HP1γ transcripts (Cbx3) following siRNA treatment measured by RT-qPCR. e, f Cell proliferation assays performed in triplicate in six-well plates. The graphs show the number of WT ESCs (e) and HP1β KO ESCs (f) treated with siRNA against HP1γ or control siRNA every 24 h. (PDF 190 kb) [file 13059_2015_760_MOESM6_ESM.pdf]

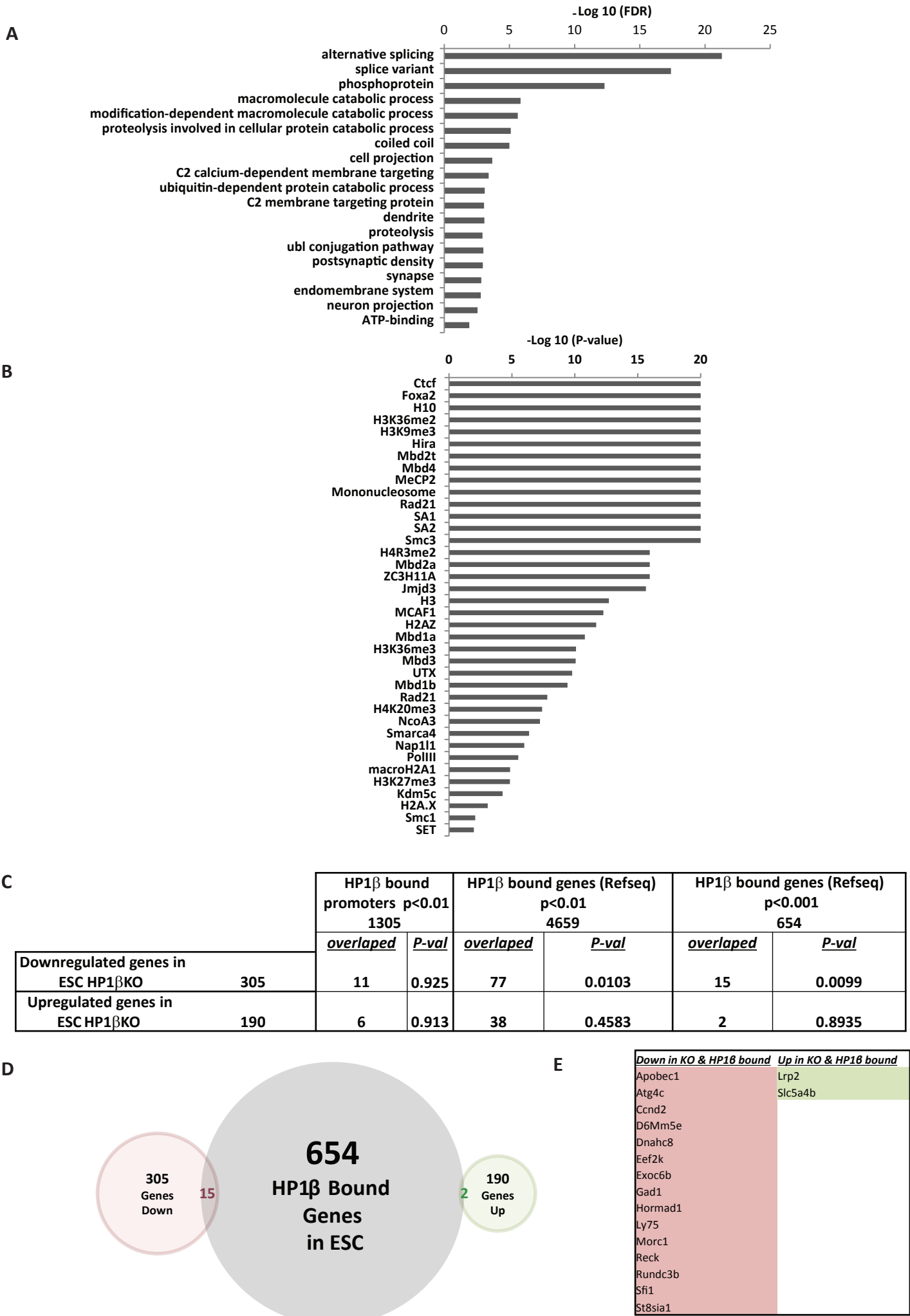

Supplement: Additional file 8: Figure S6. — HP1β ChIP-Seq analysis. a Enriched categories in Gene Ontology (GO) analysis performed for the HP1β-bound genomic regions. b Correlation analysis of HP1β ChIP-Seq data with other existing genome-wide datasets in mouse ESCs. c, d Correlation analysis of the upregulated and downregulated genes in HP1β KO ESC samples compared to WT, with HP1β-bound promoters or HP1β-bound gene bodies in WT ESC samples. e List of downregulated and upregulated genes in HP1β KO ESC samples which are also genomically bound by HP1β in WT cells. (PDF 1.19 mb) [file 13059_2015_760_MOESM8_ESM.pdf]
